# Cardio‐microcurrent device treatment for heart failure with reduced ejection fraction: Results from the C‐MIC II open‐label randomized controlled trial

**DOI:** 10.1002/ejhf.3763

**Published:** 2025-07-15

**Authors:** Jesus E. Rame, Jan D. Schmitto, Dragana N. Kosevic, Tamara Kovacevic‐Preradovic, Sasko Jovev, Marija Zdravkovic, Nermir Granov, Tanja Popov, Igor Rudez, Petar Vukovic, Velibor Ristic, Petr Neuzil, Annette Holtdirk, Arjang Ruhparwar, Muhammad Shahzeb Khan, Hans‐Dirk Düngen, Kersten Brandes, Peter Goettel, Johannes Mueller, Faouzi Kallel, Tim Friede, Miodrag Peric, Marat Fudim, Stefan D. Anker, for the C‐MIC II Trial Investigators

**Affiliations:** ^1^ Bruce and Robbi Toll Heart and Vascular Institute Thomas Jefferson University Philadelphia PA USA; ^2^ Department of Cardiac, Thoracic, Transplantation and Vascular Surgery Hannover Medical School Hannover Germany; ^3^ Cardiovascular Institute Dedinje Belgrade Serbia; ^4^ Faculty of Medicine University of Banja Luka Banja Luka Bosnia and Herzegovina; ^5^ University Clinic of Cardiology Skopje North Macedonia; ^6^ University Hospital Medical Center Bezanijska Kosa Belgrade Serbia; ^7^ Clinical Center University of Sarajevo Sarajevo Bosnia and Herzegovina; ^8^ Institute of Cardiovascular Diseases of Vojvodina Vojvodina Serbia; ^9^ University Hospital Dubrava Zagreb Croatia; ^10^ Medical School University Belgrade Belgrade Serbia; ^11^ Cardiology Clinic Medical Faculty of Charles University and Homolka Hospital Prague Prague Czech Republic; ^12^ RQM+ Frankfurt Germany; ^13^ Baylor Scott and White Health‐Heart Hospital Plano and Baylor Scott and White Research Institute Dallas TX USA; ^14^ SCIRENT LLC Berlin Germany; ^15^ Berlin Heals GmbH Berlin Germany; ^16^ Department of Medical Statistics University Medical Center Göttingen, and DZHK (German Center for Cardiovascular Research), Partner Site Lower Saxony Göttingen Germany; ^17^ Duke University Durham NC USA; ^18^ Duke Clinical Research Institute Durham NC USA; ^19^ Department of Cardiology (CVK) of German Heart Center Charité; German Centre for Cardiovascular Research (DZHK) Partner Site Berlin Charité Universitätsmedizin Berlin Germany

**Keywords:** Heart failure, Electrical microcurrent, Cardio‐microcurrent device

## Abstract

**Aims:**

In patients with heart failure, alterations in electrical fields generated within the myocardium have been associated with myocardial oedema which can act as a substrate for left ventricular dysfunction. Safety and efficacy of a direct microcurrent therapy using an implanted generator (C‐MIC) remain uncertain.

**Methods and results:**

Ambulatory patients with non‐ischaemic dilated cardiomyopathy with left ventricular ejection fraction (LVEF) of 25% to 35% and New York Heart Association (NYHA) class III–IV were randomized to C‐MIC (device) or control group in addition to guideline‐directed medical therapy. The primary endpoint was change in LVEF at 6 months. Pre‐specified secondary endpoints included 6‐min walk distance (6MWD), Kansas City Cardiomyopathy Questionnaire overall summary score (KCCQ‐OSS), and NYHA functional class. Of 70 patients randomized, 65 were included in modified intention‐to‐treat analysis (C‐MIC device: *n* = 32; control: *n* = 33). At 6 months, treatment with C‐MIC versus control improved LVEF (mean difference: 5.1%; 95% confidence interval [CI] 3.1–7.1%, *p* < 0.001). The proportions of patients with improvement in at least one NYHA class (risk difference: 68.9%; 95% CI 50.6–87.2, *p* < 0.001), an increase of ≥5 points in KCCQ‐OSS (risk difference: 60.0%; 95% CI 42.3–77.6, *p* < 0.001), and an increase of ≥30% in 6MWD (risk difference: 38.3%; 95% CI 14.4–62.2) were substantially higher in the device versus control group (*p* < 0.002).

**Conclusions:**

In patients with non‐ischaemic chronic heart failure with reduced ejection fraction, the C‐MIC device compared with control improved LVEF, symptoms, functional capacity and quality of life.

## Introduction

Chronic heart failure is associated with high morbidity and mortality rates.^1^, ^2^ Guideline‐directed medical therapy for patients with heart failure with reduced ejection fraction improves clinical outcomes with many patients achieving clinically significant recovery of myocardial function.^3^ However, often patients with heart failure with reduced ejection fraction do not achieve myocardial function recovery and continue to have high residual risk with worsening symptoms.^4^

Recent advancements have led to the development of various device‐based therapies that could potentially improve clinical outcomes and quality of life for patients with heart failure with reduced ejection fraction.^5^, ^6^, ^7^, ^8^ One such novel device is the C‐MIC device, which delivers a non‐stimulatory direct electrical current (DC; in the μA range; hence microcurrent) directly to the myocardium through a surgical implant.^9^, ^10^, ^11^ The microcurrent technology applied by the C‐MIC device has been demonstrated to enhance tissue healing based on a premise that maintaining the physiological endogenous steady potential gradient within cells is essential for proper organ function.^11^, ^12^, ^13^, ^14^ In chronic heart failure, a disruption in cardiac lymphatic drainage and alteration in electrical fields has been associated with myocardial oedema which can act as a substrate for left ventricular dysfunction.^15^, ^16^

The initial feasibility of the C‐MIC device was assessed in a single‐arm, pilot study.^11^ After 6 months, 10 patients with heart failure on maximally tolerated medical therapy who had the C‐MIC device implanted had a mean increase of 8% (absolute) in left ventricular ejection fraction (LVEF).^11^ These patients also experienced increases in 6‐min walk distance (6MWD) and quality of life. In a subsequent 2‐year follow‐up study of seven out of these 10 patients after C‐MIC deactivation, sustained improvements in LVEF, 6MWD, and quality of life were observed, with only one patient requiring reactivation of the C‐MIC device.^17^ Herein, we report the results of the first randomized controlled trial to assess the safety and efficacy of the C‐MIC device in patients with refractory ambulatory non‐ischaemic heart failure with a reduced ejection fraction.

## Methods

### Trial design and oversight

C‐MIC II is a multicentre randomized open‐label clinical trial aimed at comparing the safety and efficacy of treating heart failure patients using the C‐MIC system in addition to medical therapy versus medical therapy alone. The trial is registered as ClinicalTrials.gov Identifier: NCT04662034. The trial protocol was designed by the principal investigators and the financial sponsor. The trial was conducted in accordance with the principles of the Declaration of Helsinki and the Good Clinical Practice Guidelines of the International Council for Harmonization. The protocol was approved by institutional review boards or ethics committees at each participating location. All patients provided written informed consent. An independent data and safety monitoring committee evaluated patient safety during the trial. The steering committee and principal investigators oversaw all aspects of the trial's execution (online supplementary *Appendix* *S1*: Section A). The principal investigators had unrestricted access to the data, prepared all drafts of the manuscript, and attest to the completeness and accuracy of the data and analyses. The manuscript was reviewed and edited by all co‐authors. Financial support for the trial was provided by Berlin Heals.

### Patients, randomization, and follow‐up

Eligibility criteria included ambulatory patients with non‐ischaemic dilated cardiomyopathy who had a LVEF between 25% and 35% and were New York Heart Association (NYHA) class III to IV despite optimal medical therapy. Patients were considered ineligible for the study if they had a known ischaemic disease aetiology of heart failure. Patients with heart failure related to structural abnormalities such as congenital or valvular disease, those who received inotropic support within 30 days prior to enrolment, or patients deemed unsuitable for the C‐MIC system due to anatomical reasons were excluded. The complete list of inclusion and exclusion criteria is shown in online supplementary *Appendix* *S1*: Section B.

Patients were randomly assigned in a 1:1 ratio to either C‐MIC system (device group) or not (control group) in addition to medical therapy. The randomization was carried out using a block randomization method, and was stratified according to site. The C‐MIC system and the implantation procedure have been described previously (see online supplementary *Appendix* *S1*: Section C). Following randomization, clinical visits were scheduled at 4 weeks, and 2, 3, 4 and 6 months for both the device and control groups (online supplementary *Table* *S1*). Patients in the device group also had a clinical visit at the time of C‐MIC system implantation and a follow‐up visit within 2 weeks after the implantation. An independent blinded echocardiographic core laboratory conducted serial echocardiographic assessments at baseline, 4 weeks, 4 months, and 6 months post‐randomization.

### Trial outcomes

The primary endpoint was the difference in the change of LVEF between the device and control groups after 6 months, as assessed by the echocardiographic core laboratory. In addition to LVEF, changes in left ventricular size were evaluated by measuring the end‐systolic and end‐diastolic diameters. The three pre‐specified efficacy‐related key secondary endpoints included the proportion of patients with at least a one class improvement in NYHA functional class from baseline to 6 months, the proportion of patients with an increase of at least five points in Kansas City Cardiomyopathy Questionnaire overall summary score (KCCQ‐OSS), and the proportion of patients with at least a 30% improvement in 6MWD. The 6MWD and NYHA class assessments were conducted by individuals who were not blinded to group assignment. The eight pre‐specified safety‐related secondary endpoints included all‐cause and cardiovascular mortality, as well as device‐related deaths, hospitalizations for any reason, for cardiovascular reasons, or device‐related hospitalizations, as well as the incidence and severity of adverse events and device malfunctions.

Additional outcome assessments related to exercise capacity endpoints, including changes in absolute peak oxygen consumption (VO_2_), in body weight‐adjusted peak VO_2_, and the VO_2_ at the anaerobic threshold from baseline to 6 months, as measured by cardiopulmonary exercise testing (CPET), were performed. A comparison of the change in N‐terminal pro‐B‐type natriuretic peptide (NT‐proBNP) from baseline to 6 months between the device and control groups was also conducted.

### Statistical analysis

The sample size calculation followed a group sequential design using a two‐sample *t*‐test to assess differences in mean LVEF between the two groups. Assuming an expected mean LVEF change of 10 ± 10% in the device group and 4 ± 10% in the control group, with an alpha level of 0.05 and statistical power exceeding 80%, 46 patients were assigned to each group across two stages (interim and final analyses). Critical values for the group sequential test were determined using an O'Brien–Fleming type design. The mixed model repeated measures (MMRM) approach employed in our analyses is more powerful than a comparison of means using a two‐sample *t*‐test; therefore the power calculation is conservative. A pilot study had demonstrated that mean LVEF changes were detectable as early as 14 days post‐implantation and sustained through 6 months.^11^ Based on these findings, the interim analysis was scheduled for when 46 patients had completed their 4‐month assessment.

The primary endpoint was analysed using the MMRM approach, incorporating baseline LVEF as a covariate as well as treatment, time, treatment‐by‐time interaction and the randomization stratification variables, specifically site, as factors. To assess whether the treatment effect varied across different patient subgroups, a predefined subgroup analysis was conducted. Interaction testing was performed by including interaction terms in the MMRM model to evaluate whether treatment effects differed significantly across subgroups.

The primary efficacy and secondary analyses were performed on the modified intention‐to‐treat (mITT) population, which included all patients from the ITT group who had available at least one post‐baseline measurement of the primary endpoint. For the device group, this required successful implantation of a functional system, while for the control group, it required completion of the baseline visit. Per‐protocol (PP) analysis included all mITT patients who adhered to the study protocol and completed the study.

The Mantel–Haenszel test, stratified by site, was used to compare proportions between groups, with treatment group differences expressed as risk differences with 95% confidence intervals (CI). The MMRM approach was applied for continuous data comparisons between treatment groups. Continuous variables are presented as mean ± standard deviation or median with interquartile range (IQR). All reported *p*‐values are two‐sided. Secondary endpoints will only be tested, if the primary hypothesis could be rejected. As no hierarchy for assessing secondary endpoints was pre‐specified, all 11 secondary endpoints were considered with Bonferroni adjustment. Statistical significance for the 11 secondary endpoints was considered present if *p* < 0.00455. All other analyses were considered exploratory. Statistical analyses were conducted using SAS software, version 9.4 (SAS Institute Inc., Cary, NC, USA).

## Results

### Patients

Between January 2021 and May 2024, 70 patients were randomized across nine sites (35 in the device arm and 35 in the control arm). The C‐MIC system was successfully implanted in 32 patients; three patients assigned to the device group withdrew from the trial before any implantation attempt. Additionally, two patients from the control group withdrew after randomization. Subsequently, 65 patients were included in the mITT analysis, as only in these patients at least one assessment of the primary endpoint was available after baseline (online supplementary *Figure* *S1*). All patients completed the 6‐month clinical visit except for one patient in the control group who was lost to follow‐up.

The baseline characteristics of patients in the two groups were similar (*Table* *1*). The mean age of all patients was 60 ± 10 years, and 71% were men. The mean baseline LVEF was 30.1 ± 3.6% for the device group and 29.5 ± 3.0% for the control group. All patients were NYHA class III, except for one patient in the device group.

**Table 1:** Baseline characteristics

Characteristics	Device group (*n* = 32)	Control group (*n* = 33)
Age (years)	58.0 ± 9.5	61.9 ± 9.6
Women, *n* (%)	9 (28)	10 (30)
Body mass index (kg/m^2^)	28.5 ± 4.5	29.7 ± 3.3
Body surface area (m^2^)	2.1 ± 0.2	2.1 ± 0.2
Type 2 diabetes mellitus, *n* (%)	4 (13)	7 (21)
Hypertension, *n* (%)	24 (75)	22 (67)
Lung disease, *n* (%)	3 (9)	8 (24)
Kidney disease, *n* (%)	3 (9)	0 (0)
GI disease, *n* (%)	1 (3)	4 (12)
NYHA class III, *n* (%)	31 (97)	33 (100)
NYHA class IV, *n* (%)	1 (3)	0 (0)
KCCQ overall score, *n* (%)	45 ± 20	40 ± 25
Family history of HF, *n* (%)	13 (41)	11 (33)
SGLT2 inhibitors, *n* (%)	12 (38)	14 (42)
Beta‐blockers, *n* (%)	31 (97)	32 (97)
RAAS inhibition, *n* (%)	31 (97)	33 (100)
Aldosterone antagonists, *n* (%)	31 (97)	33 (100)
Diuretics, *n* (%)	32 (100)	33 (100)
Duration since initial HF diagnosis (years)	2.7 ± 1.5	2.8 ± 1.4
QRS duration (ms)	109 ± 19	116 ± 27
AF/atrial flutter, *n* (%)	12 (38)	11 (33)
AV block, *n* (%)	3 (9)	4 (12)
LVEF (%)	30.1 ± 3.6	29.5 ± 3.0
LVEDD (mm)	61.1 ± 6.2	62.3 ± 6.0
LVESD (mm)	52.5 ± 6.5	54.2 ± 6.6
ICD, *n* (%)	2 (6)	2 (6)
eGFR (ml/min/1.73 m^2^)	78 ± 26	78 ± 22
6MWD (m)	304 ± 63	279 ± 59
CPET, *n* (%)	32 (100)	29 (88)
Peak VO_2_ (ml/kg/min)	16 ± 5	15.5 ± 4
Exercise time (min)	8 ± 3	8 ± 3
Blood pressure (mmHg)		
Systolic	122 ± 11	121 ± 13
Diastolic	79 ± 9	77 ± 8
NT‐proBNP (pg/ml)	1153 ± 1376 Median (IQR): 684 (268–1386)	1233 ± 1062 Median (IQR): 1048 (404–1626)
Haemoglobin (g/L)	141 ± 16	149 ± 13

Data are expressed as mean ± standard deviation, unless otherwise indicated.

There were no significant between‐group differences in the characteristics at baseline.

6MWD, 6‐min walk distance; AF, atrial fibrillation; AV, atrioventricular; CPET, cardiopulmonary exercise test; eGFR, estimated glomerular filtration rate; GI, gastrointestinal; HF, heart failure; ICD, implantable cardioverter‐defibrillator; KCCQ, Kansas City Cardiomyopathy Questionnaire; LVEDD, left ventricular end‐diastolic diameter; LVEF, left ventricular ejection fraction; LVESD, left ventricular end‐systolic diameter; NT‐proBNP, N‐terminal pro‐B‐type natriuretic peptide; NYHA, New York Heart Association; RAAS, renin–angiotensin–aldosterone system; SGLT2, sodium–glucose co‐transporter 2; VO_2_, oxygen consumption.

### Primary endpoint

On 12 October 2023, the sponsor was informed by the independent data monitoring committee that criteria for overwhelming efficacy had been met and stopping the study was recommended. The data monitoring committee had analysed data of 48 patients with at least 4‐month follow‐up. At that time (October 2023), 60 patients had already been randomized. However, enrolment of participants was not stopped immediately in order to conclude co‐medication analyses (to ensure a balanced number of participants using optimal heart failure medications in both groups) as well as to arrange for regulatory discussions, which took place on 22 May 2024. Subsequent to this meeting, all sites were informed that enrolment had ended on 6 June 2024. At that time, a total of 94 patients had been enrolled, of whom 70 had been randomized in the trial (online supplementary *Figure* *S1*).

**Figure 1:**
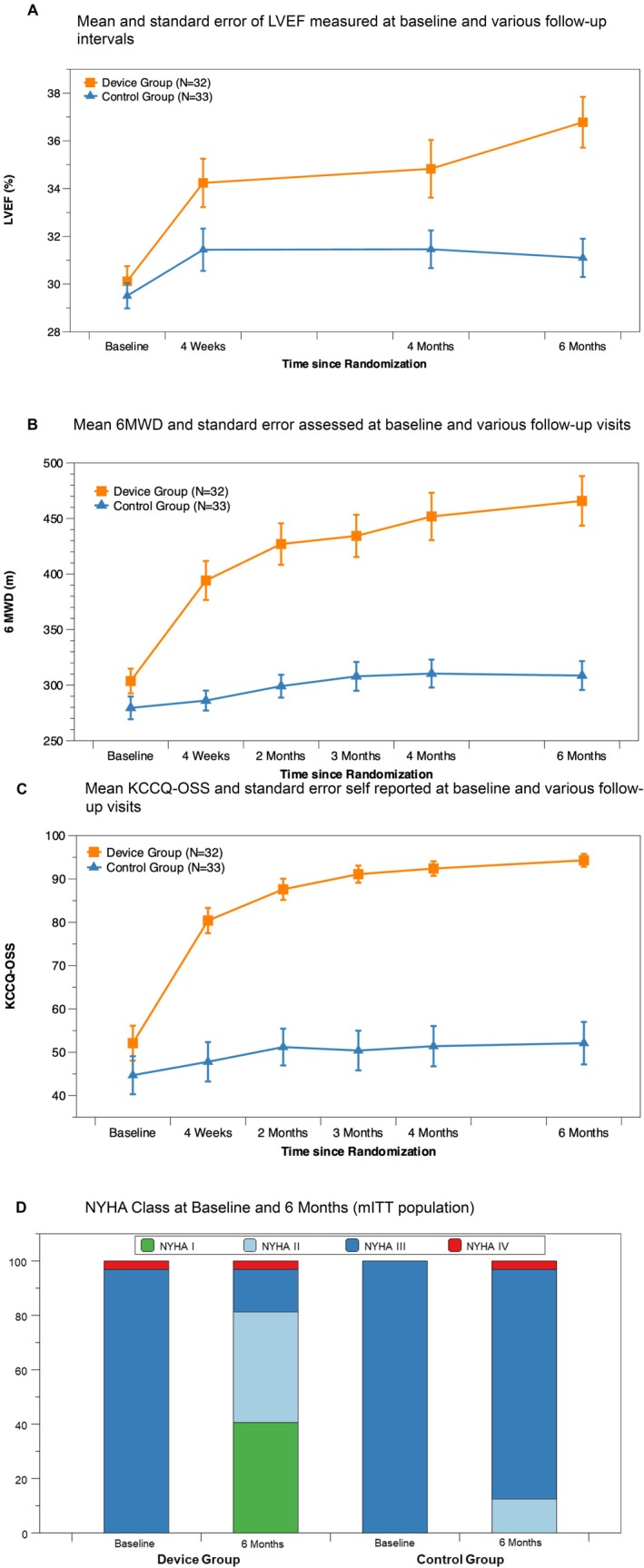
Mean and standard errors of (*A*) left ventricular ejection fraction (LVEF), (*B*) 6‐min walk distance (6MWD), (*C*) Kansas City Cardiomyopathy Questionnaire overall summary score (KCCQ‐OSS) at baseline and to various follow‐up intervals, and (*D*) New York Heart Association (NYHA) class at baseline and 6 months (modified intention‐to‐treat population).

**Table 2:** Primary and secondary efficacy and safety endpoints (modified intention‐to‐treat population)

End point	Device group (*n* = 32)	Control group (*n* = 33)	Difference (95% CI)	*p*‐value
Primary endpoint				
Change in LVEF from baseline to 6 months	6.6 ± 0.7	1.5 ± 0.7	5.1 (3.1, 7.1)	<0.001
Key (efficacy‐related) secondary endpoints				
Percentage of patients with improvement in NYHA class by more than one class at 6 months^a^	26 (81.3)	4 (12.5)	68.9% (50.6%, 87.2%)	<0.001
Percentage of patients with change in KCCQ‐OSS by ≥5 points at 6 months	31 (96.9)	16 (50.0)	60.0% (42.3%, 77.6%)	<0.001
Percentage of patients with increase in 6MWT by at least 30% at 6 months	20 (64.5)	9 (29.0)	38.3% (14.4%, 62.2%)	0.0017
Safety‐related secondary endpoints				
All‐cause death	0 (0.0)	0 (0.0)		–
Cardiac‐related death	0 (0.0)	0 (0.0)		–
Device‐related death	0 (0.0)	N/A		–
All‐cause hospitalizations	5 (15.6)	1 (3.0)	12.2% (−1.7%, 26.1%)	0.085
Cardiac‐related hospitalizations	2 (6.3)	0 (0.0)	6.4% (−2.1%, 14.9%)	0.140
Device‐related hospitalizations	0 (0.0)	N/A		–
Number of patients with serious adverse events^b^	5 (15.6)	1 (3.0)	12.6% (−1.3%, 26.5%)	0.079
Rate and number of device malfunctions associated with clinical events	0 (0)	N/A		–

Data are expressed as least‐squares mean ± standard error, or *n* (%). Efficacy analyses were conducted in the modified intention‐to‐treat population which included all randomized patients who had at least one post‐baseline measurement of the primary endpoint. For the device group, this required successful implantation of a functional system, while for the control group, it required completion of the baseline visit. Comparison between groups was performed using the Cochran–Mantel–Haenszel stratified by study sites.

6MWT, 6‐min walk test; CI, confidence interval; KCCQ‐OSS, Kansas City Cardiomyopathy Questionnaire overall summary score; LVEF, left ventricular ejection fraction; N/A, not available; NYHA, New York Heart Association.

^a^ The proportion estimate for the control group is based on 32 patients, as one patient dropped out after the 3‐month visit.

^b^ In the device group, serious adverse events included hemothorax in two patients, worsening renal function in one patient, pericardial effusion in one patient, a single episode of hypotension in one patient, and a single episode of ventricular arrhythmia in one patient. In the control group, one patient experienced a leg fracture.

**Figure 2:**
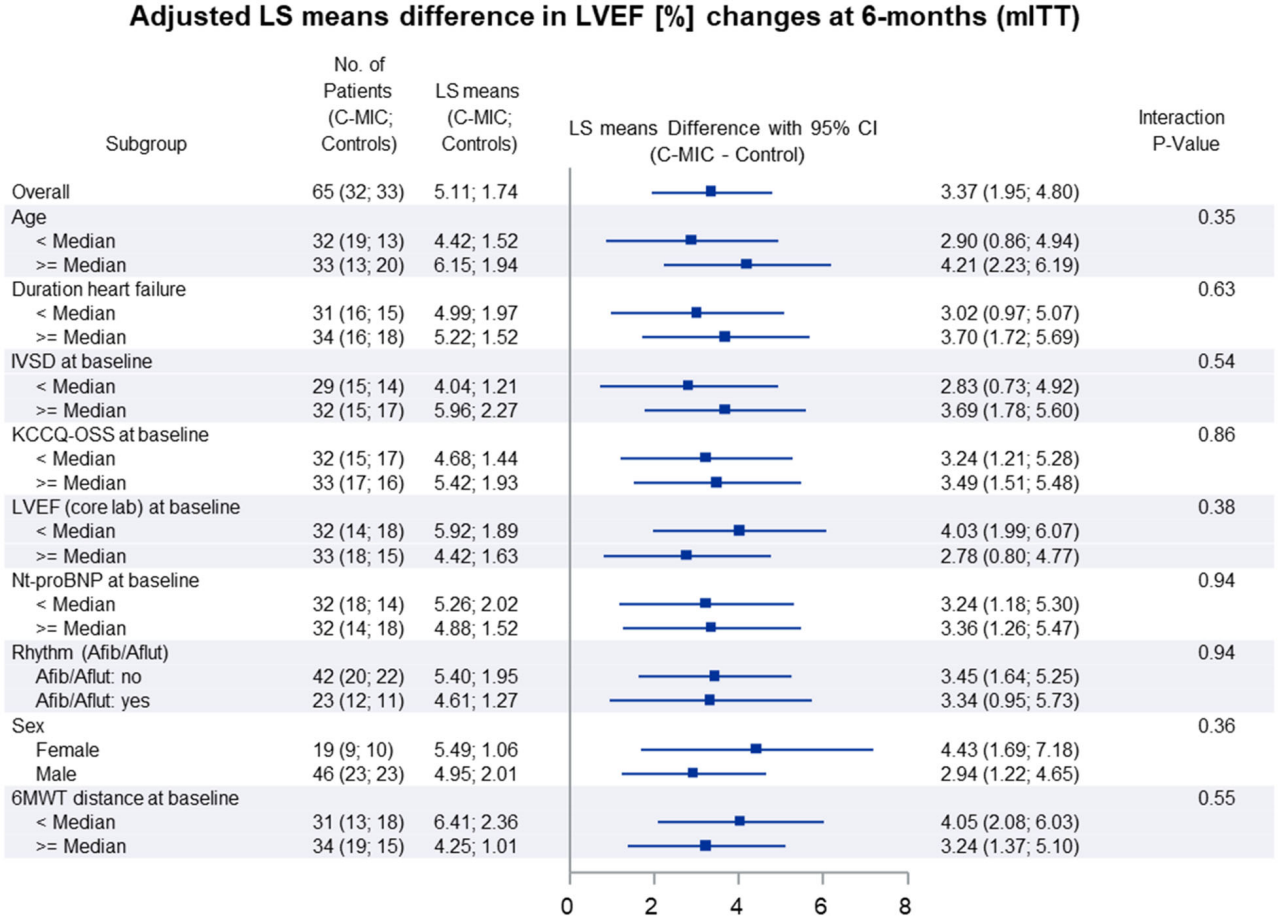
Sub‐group analysis of the primary endpoint based on the modified intention‐to‐treat (mITT) analysis. 6MWT, 6‐min walk test; CI, confidence interval; C‐MIC, cardio‐microcurrent device; IVSD, interventricular septal end‐diastole; KCCQ‐OSS, Kansas City Cardiomyopathy Questionnaire overall summary score; LS, least squares; LVEF, left ventricular ejection fraction; Nt‐proBNP, N‐terminal pro‐B‐type natriuretic peptide.

*Figure* *1A* shows the mean LVEF with standard error at each follow‐up visit for both the device and control groups, highlighting the temporal pattern of LVEF. The mean change from baseline in LVEF at 6 months was 6.6% (95% CI 5.2–8.0) in the device group and 1.5% (95% CI 0.08–2.9) in the control group (*Table* *2*). The estimated treatment difference was 5.1% (95% CI 3.1–7.1, *p* < 0.001) (*Graphical Abstract*). Sub‐group analyses of the primary endpoint at 6 months showed no heterogeneity of treatment effect in any of the subgroups (*Figure* *2*).

Of note, at 6 months, the mean change from baseline in left ventricular end‐diastolic diameter was −1.1 mm (95% CI −2.9 to +0.8) in the device group and −1.2 mm (95% CI −3.1 to +0.7) in the control group (*p*>0.4 between groups). The mean change in left ventricular end‐systolic diameter was −2.9 mm (95% CI −5.0 to −0.9) in the device group and −1.7 mm (95% CI −4.7 to +1.1) in the control group (all *p*>0.4 between groups).

### Secondary endpoints

*Figure* *1B* presents 6MWD with standard error at each follow‐up visit for both the device and control groups, illustrating functional improvement over time. The mean change in 6MWD at 6 months was 165 ± 18 m in the device group and 20 ± 18 m in the control group (*Table* *3*). The estimated treatment difference was 144.7 m (95% CI 93.8–195.5, *p* < 0.001) (*Graphical Abstract*). The proportion of patients achieving a 30% increase in 6MWD was higher in the device group compared to the control group, with a risk difference of 38.3% (95% CI 14.4–62.2, *p* = 0.0017) (*Table* *2*). Similarly, the risk difference for patients achieving at least a 30‐m increase in 6MWD was 39.3% (95% CI 13.1–65.5, *p* = 0.0033).

**Table 3:** Further endpoints (modified intention‐to‐treat population)^a^

	Device group (*n* = 32)	Control group (*n* = 33)	Risk difference (95% CI)	*p*‐value
Further clinical assessments				
Change in NYHA class from baseline to 6 months^c^	1.15 ± 0.11	0.003 ± 0.11	1.14 (0.82 to 1.47)	<0.001
Change in 6MWD from baseline to 6 months^c^ (m)	165.0 ± 18.1	20.3 ± 18.0	144.7 (93.8 to 195.6)	<0.001
Percentage of patients with 6MWD increase by ≥30 m from baseline to 6 months^b^	27 (87.1)	17 (54.8)	39.3% (13.1%, 65.5%)	0.0033
Change in KCCQ‐OSS from baseline to 6 months^c^	46.4 ± 3.3	5.7 ± 3.1	40.7 (31.9 to 49.5)	<0.001
Percentage of patients with change in KCCQ‐OSS by ≥10 points at 6 months^b^	29 (90.6)	13 (40.6)	57.2% (38.1%, 76.2%)	<0.001
Percentage of patients with change in KCCQ‐OSS by ≥15 points at 6 months^b^	28 (87.5)	9 (28.1)	61.2% (42.0%, 80.4%)	<0.001
Exercise testing results				
Change in peak VO_2_ from baseline to 6 months^c^ (ml/min)	114.5 ± 63.1	−17.9 ± 66.2	132.4 (−49.8 to 314.5)	0.150
Change in body weight‐adjusted peak VO_2_ from baseline to 6 months^c^ (ml/kg/min)	1.8 ± 0.7	−0.26 ± 0.76	2.03 (−0.06 to 4.12)	0.056
Change in VO_2_ AT from baseline to 6 months^c^ (ml/min)	−4.2 ± 90.9	−4.2 ± 96.6	−0.05 (−269.8 to 269.7)	0.99
Change in body weight‐adjusted VO_2_ AT from baseline to 6 months^c^ (ml/kg/min)	0.16 ± 0.96	−0.30 ± 1.02	0.46 (−2.39 to 3.30)	0.71
Change in exercise duration from baseline to 6 months^c^ (min)	1.56 ± 0.60	0.51 ± 0.63	1.05 (−0.69 to 2.79)	0.23

6MWD, 6‐min walk distance; AT, anaerobic threshold; CI, confidence interval; KCCQ‐OSS, Kansas City Cardiomyopathy Questionnaire overall summary score; NYHA, New York Heart Association; VO_2_, oxygen consumption.

^a^ Efficacy analyses were conducted in the modified intention‐to‐treat population which included all randomized patients who had at least one post‐baseline measurement of the primary endpoint. For the device group, this required successful implantation of a functional system, while for the control group, it required completion of the baseline visit.

^b^ Comparison between groups was performed using the Cochran–Mantel–Haenszel stratified by study sites. The proportion estimate for the control group is based on 32 patients, as one patient dropped out after the 3‐month visit.

^c^ Comparison between groups was based on the mixed effect of multiple measurements method. Peak VO_2_ data were available for 31 of 32 patients in the device group and 28 of 33 in the control group. VO_2_ AT was available for 25 of 32 patients in the device group and 21 of 33 in the control group.

*Figure* *1C* depicts the mean KCCQ‐OSS with standard error at each follow‐up visit for both the device and control groups, illustrating the trajectory of patient‐reported health status improvement over time. The mean change in KCCQ‐OSS at 6 months was 46.4 ± 3.3 in the device group compared to 5.7 ± 3.1 in the control group, resulting in a difference of 40.7 points (95% CI 31.9–49.5, *p* < 0.001) (*Graphical Abstract*). The risk differences in the proportion of patients achieving an increase of more than 5, 10, and 15 points from baseline to 6 months were 60.0% (95% CI 42.3–77.6, *p* < 0.001), 57.2% (95% CI 38.1–76.2, *p* < 0.001), and 61.2% (95% CI 42.0–80.4, *p* < 0.001), respectively (*Table* *3*).

More patients in the device group (*n* = 26; 81%) improved by at least one NYHA class from baseline, compared to the control group (*n* = 4; 12%) (risk difference: 68.9%, 95% CI 50.6–87.2, *p* < 0.001). At 6 months, 81.2% of patients in the device group were in NYHA class I/II, compared to only 12.5% in the control group (*p* < 0.001) (*Figure* *1D*).

Cardiopulmonary exercise testing results reported in *Table* *3* show a trend toward a greater increase in peak VO_2_ in the device group compared to the control group, with a mean difference of +2.03 ml/kg/min (95% CI −0.06 to 4.12; *p* = 0.056) (online supplementary *Figure* *S2*). While this difference did not reach statistical significance, the directional change was consistent with other functional outcomes. In contrast, there was no observed difference between groups in the change in VO_2_ at the anaerobic threshold. Approximately 30% of VO_2_ at the anaerobic threshold data were missing in both groups, which may have limited the power to detect a meaningful difference.

**Figure 3:**
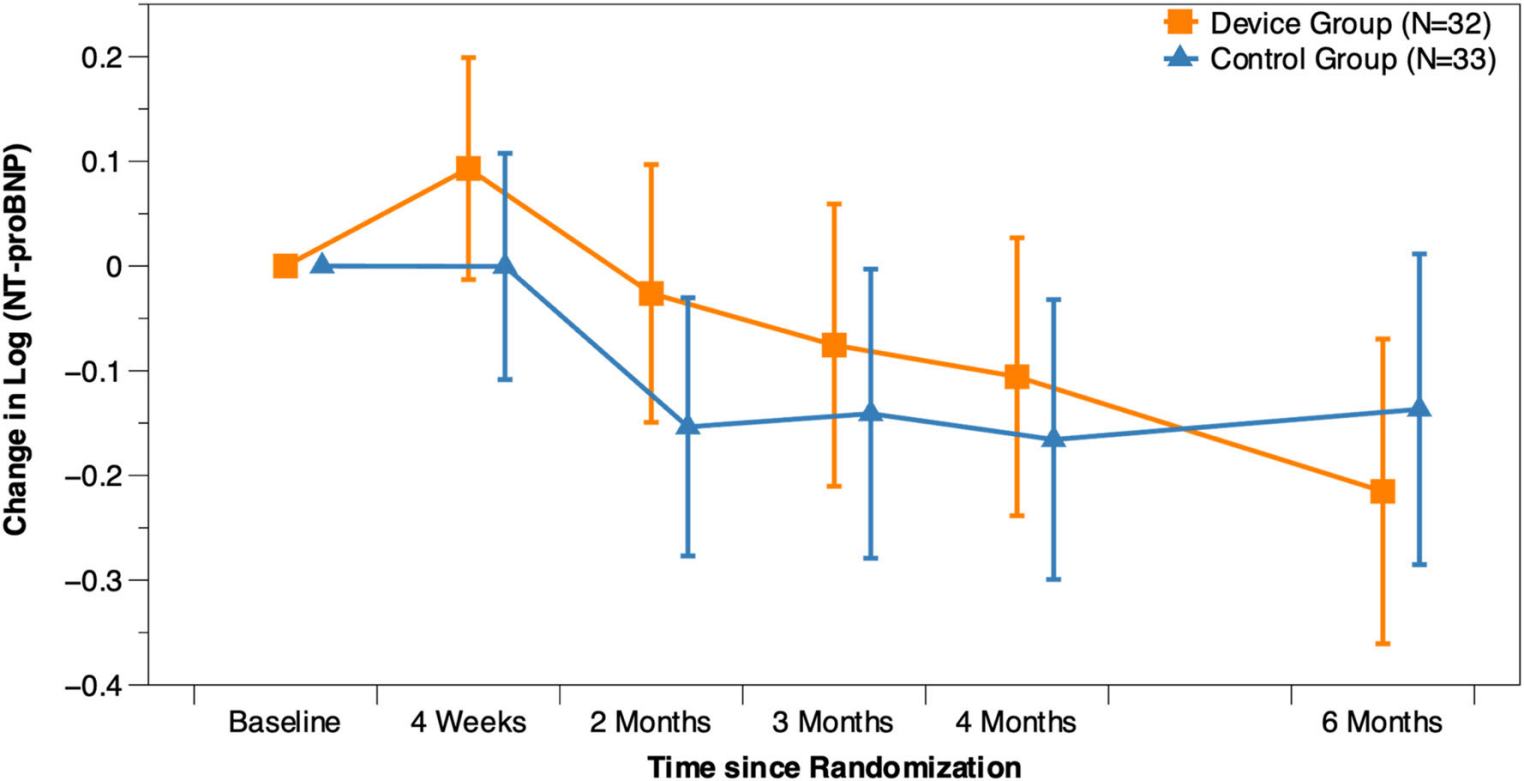
Change in N‐terminal pro‐B‐type natriuretic peptide (NT‐proBNP) from baseline to 6 months (log scale). Note that a difference on the log scale translates to a ratio on the original scale. For example, a log‐scale difference of −0.0784 between the device and control groups at 6 months corresponds to a 7.5% reduction in NT‐proBNP on the original scale.

The comparison of changes in NT‐proBNP between the device and control groups is presented in *Figure* *3*. Distinct temporal trends were observed; in the device group, NT‐proBNP levels initially rose within the first 4 weeks post‐implantation, then steadily declined over the 6‐month follow‐up. In contrast, the control group exhibited a modest decrease around 2 months post‐enrolment, with levels remaining relatively stable thereafter (*Table* *4*).

**Table 4:** Safety data (per protocol population)^a^

	Device group (*n* = 30)	Control group (*n* = 29)	Risk difference (95% CI)	*p*‐value
All‐cause death, *n* (%)	0 (0)	0 (0.0)		
All hospitalization, *n* (%)	4 (13.3)	0 (0.0)	13.3% (1.2%, 25.5%)	0.041
Cardiac‐related hospitalization, *n* (%)	1 (3.3)	0 (0.0)	3.3% (−3.1%, 9.8%)	0.32
Device‐related hospitalization, *n* (%)	0 (0)	N/A		
Surgery‐related hospitalization, *n* (%)	3 (10)	N/A		
Serious adverse events, *n* (%)	4 (13.3)^b^	0 (0.0)	13.3% (1.2%, 25.5%)	0.041

CI, confidence interval; N/A, not available.

^a^ Safety analyses were conducted in the per protocol population which included all patients included in the mITT and who adhered to the study protocol without major violations and completed the study. Comparison between groups was performed using the Cochran–Mantel–Haenszel stratified by study sites.

^b^ Hemothorax in two patients, worsening of renal function in one patient, pericardial effusion in one patient, and one episode of hypotension in one patient.

Blood pressure was adequately controlled in both the device and control groups throughout the study period. Systolic and diastolic blood pressure values remained stable over time in both arms (online supplementary *Figure* *S3*). A mixed model for repeated measures analysis confirmed that there were no significant differences in the changes in either systolic or diastolic blood pressure from baseline to 6 months between the two groups.

### Safety

The C‐MIC system was successfully implanted in all patients, with a mean skin‐to‐skin procedure time of 2.4 ± 0.9 h. According to the surgical notes, the epicardial patch was implanted outside the pericardial space in 30 patients and within the pericardial sac in 2 patients. The median hospital length of stay was 5 days (IQR 5–7). There was no mortality reported. A total of 28 adverse events were reported (6 events in the control group and 22 in the device group). Serious adverse events (SAEs) were seen in six patients (five in the device group vs. one in the control group). SAEs included hemothorax in two patients, pleural effusion in the left hemithorax in another, and a leg fracture in the control group. The rate of all hospitalizations was higher in the device group with a risk difference of 12.2% (95% CI −1.7% to 26.1%). Among all hospitalizations, only two were cardiac related. A detailed list of adverse events is shown in online supplementary *Tables* *S2* and *S3*. Non‐SAEs were reported in 11 patients in the device group, including wound infection, COVID‐19, knee pain, and dizziness. In the control group, non‐SAEs were reported in five patients and included fatigue, shortness of breath, and respiratory infection (online supplementary *Table* *S3*). Statistical testing for all safety outcomes of the mITT population showed *p* > 0.05 for all assessments. While device‐related deficiencies were reported, none were linked to clinical adverse events or interruptions in therapy delivery (online supplementary *Table* *S4*).

## Discussion

In this first randomized trial of cardiac microcurrent device therapy for chronic heart failure, we found that the C‐MIC device compared with the control group provided improvement in LVEF at 6 months. Improvement was also observed in 6MWD, KCCQ‐OSS and NYHA functional class at 6 months in the device group compared with the control group. The device was overall well tolerated and was considered safe within this study with no mortality reported. These findings are important as the C‐MIC device could provide an important treatment option for patients with non‐ischaemic chronic heart failure with reduced ejection fraction who do not achieve myocardial function recovery and continue to have worsening symptoms despite maximally tolerated medical therapy.

In a prior pilot study, C‐MIC had demonstrated an increase in LVEF as early as 2 weeks after implantation, which was sustained up to 6 months.^11^ Myocardial recovery is a clinically important, patient centred outcome in patients with chronic dilated cardiomyopathy.^18^ Long‐term outcomes of patients who have demonstrated some degree of restoration of cardiac function, transitioning to heart failure with recovered ejection fraction are significantly better.^19^, ^20^ Given that the improvement in LVEF was not associated with significant changes in left ventricular dimensions in our study, we consider this to be suggestive of improved left ventricular contractility during the 6‐month treatment period. Along with the observation of early improvement in LVEF (within 4 weeks) the absence during this period of left ventricular reverse remodelling, which is often observed in months, suggests a novel yet to be determined mechanism of microcurrent delivery to the myocardium. Whether longer‐term therapy would result in reverse remodelling, remains to be studied. The results from this study prompt further investigation not only into the mechanisms which subtend the early and sustained changes in myocardial function, but also into the application of microcurrent device therapy to a broader population of patients with heart failure, including with ischaemic disease aetiology and those with devices.

While the enrolled patients demonstrated key indicators of advanced heart failure—including NYHA class III symptoms, LVEF <35%, elevated NT‐proBNP levels, and reduced 6MWD—the limited use of implantable cardioverter‐defibrillators (ICD) in this cohort reflects the specific clinical profile of the study population. All participants had non‐ischaemic dilated cardiomyopathy, for which the European Society of Cardiology guidelines provide a Class IIa recommendation for ICD implantation, as opposed to the stronger Class I recommendation in ischaemic heart failure. Moreover, most patients had narrow QRS durations, limiting eligibility for cardiac resynchronization therapy (CRT)‐based device therapies. Consequently, only two patients in each arm had received an ICD. The observed preservation of kidney function and relatively low event rates likely stem from careful patient selection, early‐stage disease in a subset of participants, and the structured follow‐up inherent to a clinical trial setting. This is also consistent with the known lower prevalence of chronic kidney disease in non‐ischaemic versus ischaemic heart failure populations.

Patients with heart failure often have poor quality of life and functional status despite medical therapy.^21^, ^22^ This trial showed that the C‐MIC device leads to improvement in 6MWD and KCCQ‐OSS on top of medical therapy. Improvement in NYHA functional class was also observed at 6 months in the device group. The observed improvements in symptoms, functional capacity, and health status with the C‐MIC device are larger compared with other existing heart failure devices or medical therapy.^23^, ^24^, ^25^ An observed improvement in KCCQ‐OSS approaching 90 in some patients may reflect, at least in part, a substantial placebo effect, which is commonly seen in heart failure trials involving device‐based interventions. However, this marked improvement in health status may also be attributable to the immediate and sustained enhancement in LVEF following C‐MIC therapy. The observed concordance between objective improvements in cardiac function and patient‐reported outcomes suggests that the clinical benefit may extend beyond placebo response alone. Considering that improving quality of life and functional status in patients with heart failure is the top most priority for many physicians and patients alike,^26^ C‐MIC may be an important future therapeutic option for a subset of patients with heart failure.

Both the device and control groups were well balanced at baseline and received optimal guideline‐directed medical therapy as detailed in *Table* *1*. Changes in heart failure medication during the 6‐month study period were minimal and comparable between groups. While the overall use of sodium–glucose co‐transporter 2 inhibitors was relatively low (~40%), this reflects the slower adoption and limited availability of these agents in several East European countries during the study period. Given the consistent use of standard heart failure therapies across both groups and the minimal changes observed over time, it is unlikely that differences in pharmacologic treatment explain the observed differences in clinical outcomes.

The observed changes in peak VO_2_ offer additional insight into the functional impact of C‐MIC therapy. Patients in the device group demonstrated a trend toward a greater increase in peak VO_2_ from baseline to 6 months compared to controls, with a borderline *p*‐value of 0.056. However, the absence of a centralized core laboratory and the lack of a standardized CPET protocol across sites may have introduced variability and limited the precision of these measurements. Importantly, while CPET provides an objective, quantitative assessment of maximal cardiorespiratory fitness, it differs in scope from other functional endpoints such as the 6‐min walk test and the KCCQ, which reflect submaximal effort and patient‐reported quality of life. The combination of these complementary measures may offer a more comprehensive view of treatment response, capturing both physiological capacity and real‐world functional improvement.

While NT‐proBNP is a well‐established biomarker of heart failure severity and prognosis, its response to microcurrent therapy has been inconsistent. In this study, despite clear and sustained improvements in symptoms, functional capacity, and echocardiographic parameters, NT‐proBNP levels did not show significant decline. One possible explanation is that myocardial wall stress remained elevated during the 6‐month observation period, which aligns with the lack of significant left ventricular end‐diastolic diameter reduction observed in this study. This suggests that microcurrent therapy may exert its benefits through mechanisms not directly associated with reductions in wall stress—particularly during the initial 6‐month treatment phase, which NT‐proBNP primarily reflects. Potential pathways may include anti‐inflammatory effects, bioelectrical modulation, or cellular remodelling, which are not adequately captured by traditional heart failure biomarkers. Additionally, variability among patients and assay limitations may contribute to the observed inconsistency. Future studies incorporating serial blood sampling and *in vitro* proteomic analyses are planned to further elucidate the therapy's mechanism of action and to help identify more specific and responsive biomarkers.

Microcurrent therapy has been successfully applied in various clinical settings, including wound healing, where it has demonstrated benefits in promoting tissue repair, reducing inflammation, and enhancing cellular regeneration.^27^, ^28^ Building on these principles, C‐MIC therapy applies low‐level direct microcurrents to myocardial tissue through an implantable device with the aim of promoting left ventricular recovery in chronic heart failure. While the precise mechanisms remain under investigation, preclinical studies suggest several plausible pathways.^12^, ^28^ Microcurrents may restore impaired bioelectric signaling, stabilize cardiomyocyte membrane potentials, and improve myocardial synchronization. Sustained effects may be driven by enhanced ATP production, increased protein synthesis, and the upregulation of angiogenic and reparative growth factors such as vascular endothelial growth factor and insulin‐like growth factor‐1.^29^ These actions may reduce fibrosis, support capillary density, and promote cardiomyocyte survival in diseased myocardium. Additionally, microcurrent‐induced electroosmosis may reduce myocardial oedema by mobilizing interstitial fluid—an effect previously linked to functional improvements in other tissues and a potentially underappreciated contributor to systolic dysfunction in dilated cardiomyopathy.^28^ Future trials incorporating advanced imaging and biomarker endpoints will be critical to confirm these mechanistic effects and refine patient selection strategies for C‐MIC therapy.

The surgical implant was well tolerated with most patients in the device group discharged between 5 and 7 days of implantation. Significant adverse events included hemothorax in two patients while there was no perioperative mortality in the device group and none of the patients required any temporary, mechanical circulatory support pre‐or post‐device implantation. The nominally higher incidence of SAEs in the device group compared to the control group was primarily related to procedural complications from the surgical placement of the epicardial patch via thoracotomy. Events such as hemothorax (occurring twice), pericardial effusion (occurring once), and transient hypotension (occurring once) are recognized risks associated with this surgical approach. These findings align with previously published data, including a study by Haight *et al*.,^30^ which reported a 5% incidence of bleeding complications following epicardial pacemaker implantation via lateral thoracotomy. Notably, none of the adverse events in our study resulted in lasting clinical harm or interfered with therapy delivery, indicating that while the procedure carries known risks, they are generally manageable with appropriate perioperative support.

It is important to highlight that in the 6‐month period of follow‐up for the pre‐specified primary endpoint there were no hospitalizations due to heart failure in the device and control groups. This period of follow‐up fell within the high prevalence interval of the COVID‐19 pandemic, which makes the results difficult to interpret as the patients and their providers in both groups may have identified alternate strategies to address heart failure decompensation.

Although the C‐MIC device is activated for only 6 months, the decision to explant the epicardial patch should be carefully weighed against the surgical risks associated with reoperation. In general, it is recommended to leave the patch in place, as device abandonment is considered a safe and common practice for inactive implantable components. The long‐term safety of leaving the C‐MIC device in situ will be further evaluated in the ongoing 2‐year follow‐up of the C‐MIC II study. The 6‐month therapy duration was informed by years of preclinical work—including cellular, small animal and large animal studies—that indicated sustained benefits from several months of treatment. However, as pioneers in this form of therapy, we have not yet conducted a detailed dose duration across multiple timeframes. Our ability to explore longer duration has also been limited by current battery capacity. In the C‐MIC I follow‐up study, we included an option to reactivate therapy during the 2‐year follow‐up period following the initial 6‐month treatment. This reactivation occurred in only one patient, but it highlights an important area for future investigation (Rame J.E., unpublished data).

### Limitations

A key limitation of this study is the potential for bias in outcome assessment. Although the echocardiographic core lab was blinded to treatment assignment, the C‐MIC lead may have been visible on some images, potentially revealing group allocation. Additionally, the assessments of 6MWD and NYHA class were conducted by personnel who were not blinded, which may have introduced observer bias. Furthermore, the notable improvements observed in patient‐reported KCCQ scores may, in part, reflect a placebo effect, which is commonly seen in device‐based trials and could have influenced patients' perception of symptom improvement. These factors should be considered when interpreting the functional, echocardiographic, and quality‐of‐life outcomes. Future trial designs will incorporate strategies to mitigate these potential sources of bias and better account for placebo effects.

Another important limitation of this study is the early termination of enrolment, which may introduce bias and potentially overestimate the treatment effect—an issue that has been documented in trials stopped early for efficacy benefit. Although the protocol and statistical analysis plan did not include provisions for stopping the trial based on efficacy, an interim analysis was pre‐specified to assess outcomes in the control group and confirm that the study remained adequately powered to meet its primary endpoint. Upon review of the unblinded data, the independent data monitoring committee observed a highly significant improvement in the primary endpoint (*p* < 0.0001), along with favourable trends across multiple secondary endpoints, and recommended stopping the study in October 2023. Recognizing the deviation from the original trial plan, the sponsor consulted the German Notified Body. During this review period, enrolment continued—from 60 patients randomized at the time of the interim analysis to 70 by trial closure. The Notified Body did not oppose the data monitoring committee recommendation, and enrolment was formally stopped on 6 June 2024. While this decision was made with careful ethical consideration and appropriate oversight, the early termination of the study may limit the generalizability of the findings and increases the risk of bias, particularly in subjective secondary endpoints.

An additional limitation is that patients with implanted cardiac devices—such as ICDs, CRTs, pacemakers, and cardiac contractility modulation systems—were excluded from this study (with the exception of ICDs with single‐coil leads) due to the potential for electrical interaction with the continuous direct microcurrent delivered by the C‐MIC system. As a result, the findings may not be generalizable to the broader heart failure population with such devices, and further testing is needed to confirm safety and compatibility.

The study population consisted of patients with a relatively short history of heart failure (1–5 years), which may have contributed to the favourable treatment response. Patients in earlier stages of disease may retain greater myocardial adaptability, allowing for more pronounced restorative effects. As such, these findings may not fully extend to patients with longer standing or more advanced heart failure, underscoring the need for further studies in broader populations.

Additionally, the trial was not powered to determine if the C‐MIC device would reduce morbidity and mortality events. However, data from recent ambulatory registry cohorts have shown improvements in LVEF in heart failure with reduced ejection fraction patients are associated with better outcomes,^31^, ^32^, ^33^, ^34^ which support the rationale for larger, event‐driven studies in the future.^32^ Furthermore long‐term data to confirm durability of the C‐MIC device are currently lacking. A 2‐year follow‐up study is currently underway (NCT05189860).

Another limitation is the absence of core laboratory analysis for CPET data which may have introduced variability in data acquisition and interpretation across sites. Data on exercise capacity at the anaerobic threshold were missing in approximately one‐third of patients, further limiting the robustness of conclusions drawn from this parameter. Dedicated studies on CPET are better suited for such outcomes than larger‐scale clinical trial focusing on general outcomes.

Finally, the trial was conducted exclusively at sites in Eastern Europe. While this may raise questions about the broader applicability of the findings, current evidence does not clearly demonstrate significant differences in heart failure phenotypes or treatment responses between Eastern and Western European populations. Nonetheless, we acknowledge that geographic, genetic, and healthcare system‐related factors may influence outcomes. Further studies involving more diverse populations are therefore needed to confirm the generalizability of these results.

## Conclusion

In conclusion, in patients with non‐ischaemic chronic heart failure with reduced ejection fraction, the C‐MIC device compared with control improved LVEF, NYHA functional class, self‐reported health status, and quality of life.

## Supporting information


**Appendix S1.** Supporting Information.

## References

[1] Tromp J , Bamadhaj S , Cleland JGF , Angermann CE , Dahlstrom U , Ouwerkerk W , *et al*. Post‐discharge prognosis of patients admitted to hospital for heart failure by world region, and national level of income and income disparity (REPORT‐HF): A cohort study. Lancet Glob Health 2020;8:e411‐e422. 10.1016/S2214-109X(20)30004-8 32087174

[2] Rosano GMC . Clinical trial design, endpoints and regulatory considerations in heart failure. Glob Cardiol 2024;2. 10.4081/cardio.2024.18

[3] Yan CL , Snipelisky D , Velez M , Baran D , Estep JD , Bauerlein EJ , *et al*. Protocol‐driven approach to guideline‐directed medical therapy optimization for heart failure: A real‐world application to recovery. Am Heart J Plus 2024;45:100438. 10.1016/j.ahjo.2024.100438 39220718 PMC11362780

[4] Wilcox JE , Fonarow GC , Ardehali H , Bonow RO , Butler J , Sauer AJ , *et al*. “Targeting the heart” in heart failure: Myocardial recovery in heart failure with reduced ejection fraction. JACC Heart Fail 2015;3:661–669. 10.1016/j.jchf.2015.04.011 26362444

[5] Mody R , Nee Sheth AB , Dash D , Mody B , Agrawal A , Monga IS , *et al*. Device therapies for heart failure with reduced ejection fraction: A new era. Front Cardiovasc Med 2024;11:1388232. 10.3389/fcvm.2024.1388232 39494238 PMC11527719

[6] Bazoukis G , Saplaouras A , Efthymiou P , Yiannikourides A , Liu T , Letsas KP , *et al*. Cardiac contractility modulation in patients with heart failure – a review of the literature. Heart Fail Rev 2024;29:689–705. 10.1007/s10741-024-10390-1 38393423

[7] Palmiero G , Florio MT , Rubino M , Nesti M , Marchel M , Russo V . Cardiac resynchronization therapy in patients with heart failure: What is new? Heart Fail Clin 2021;17:289–301. 10.1016/j.hfc.2021.01.010 33673953

[8] Antoniadis AP , Sieniewicz B , Gould J , Porter B , Webb J , Claridge S , *et al*. Updates in cardiac resynchronization therapy for chronic heart failure: Review of multisite pacing. Curr Heart Fail Rep 2017;14:376–383. 10.1007/s11897-017-0350-z 28779280

[9] Kapeller B , Mueller J , Losert U , Podesser BK , Macfelda K . Microcurrent stimulation promotes reverse remodelling in cardiomyocytes. ESC Heart Fail. 2016;3:122–130. 10.1002/ehf2.12080 27774272 PMC5064659

[10] Schmitto JD , Napp LC , Mariani S , Hanke JS , Li T , Vogel‐Claussen J , *et al*. First‐in‐man implantation of a cardiac microcurrent device for chronic systolic heart failure. ASAIO J 2022;68:e121–e123. 10.1097/MAT.0000000000001537 34324448

[11] Kosevic D , Wiedemann D , Vukovic P , Ristic V , Riebandt J , Radak U , *et al*. Cardio‐microcurrent device for chronic heart failure: First‐in‐human clinical study. ESC Heart Fail 2021;8:962–970. 10.1002/ehf2.13242 33559358 PMC8006737

[12] Bachamanda Somesh D , Jürchott K , Giesel T , Töllner T , Prehn A , Richters JP , *et al*. Microcurrent‐mediated modulation of myofibroblasts for cardiac repair and regeneration. Int J Mol Sci 2024;25:3268. 10.3390/ijms25063268 38542242 PMC10970173

[13] Rouabhia M , Park H , Meng S , Derbali H , Zhang Z . Electrical stimulation promotes wound healing by enhancing dermal fibroblast activity and promoting myofibroblast transdifferentiation. PLoS One 2013;8:e71660. 10.1371/journal.pone.0071660 23990967 PMC3747189

[14] Avendaño‐Coy J , López‐Muñoz P , Serrano‐Muñoz D , Comino‐Suárez N , Avendaño‐López C , Martin‐Espinosa N . Electrical microcurrent stimulation therapy for wound healing: A meta‐analysis of randomized clinical trials. J Tissue Viability 2022;31:268–277. 10.1016/j.jtv.2021.12.002 34903470

[15] Pu Z , Shimizu Y , Hayashi T , Che Y , Suzuki J , Tsuzuki K , *et al*. Cardiac lymphatic insufficiency leads to diastolic dysfunction via myocardial morphologic change. JACC Basic Transl Sci 2023;8:958–972. 10.1016/j.jacbts.2023.01.008 37719433 PMC10504400

[16] Kiseleva DG , Kirichenko TV , Markina YV , Cherednichenko VR , Gugueva EA , Markin AM . Mechanisms of myocardial edema development in CVD pathophysiology. Biomedicines 2024;12:465. 10.3390/biomedicines12020465 38398066 PMC10887157

[17] Kosevic DB , Radak U , Vukovic P , Schmitto JD , Brandes K , Goettel P , *et al*. Two‐year outcomes of a cardiac microcurrent device in chronic heart failure: A first‐in‐human pilot study. ESC Heart Fail 2025. 10.1002/ehf2.15369 PMC12450836 40634236

[18] Pensa AV , Zheng V , Davis L , Harap RW , Wilcox JE . Clinical perspective of myocardial recovery and improvement: Definitions, prevalence, and relevance. Methodist Debakey Cardiovasc J 2024;20:6–15. 10.14797/mdcvj.1441 PMC11342833 39184164

[19] Park CS , Park JJ , Mebazaa A , Oh IY , Park HA , Cho HJ , *et al*. Characteristics, outcomes, and treatment of heart failure with improved ejection fraction. J Am Heart Assoc 2019;8:e011077. 10.1161/JAHA.118.011077 30845873 PMC6475046

[20] Sun Y , Chen X , Zhang Y , Yu Y , Zhang X , Si J , *et al*. Reverse atrial remodeling in heart failure with recovered ejection fraction. J Am Heart Assoc 2023;12:e026891. 10.1161/JAHA.122.026891 36645090 PMC9939067

[21] Khan MS , Butler J , Friede T , Ponikowski P , Anker SD . To clip or not to clip moderate‐to‐severe functional mitral regurgitation in patients with heart failure. Glob Cardiol 2024;2. 10.4081/cardio.2024.44

[22] Oskouie S , Pandey A , Sauer AJ , Greene SJ , Mullens W , Khan MS , *et al*. From hospital to home: Evidence‐based care for worsening heart failure. JACC Adv 2024;3:101131. 10.1016/j.jacadv.2024.101131 39184855 PMC11342447

[23] Khariton Y , Fonarow GC , Arnold SV , Hellkamp A , Nassif ME , Sharma PP , *et al*. Association between sacubitril/valsartan initiation and health status outcomes in heart failure with reduced ejection fraction. JACC Heart Fail 2019;7:933–941. 10.1016/j.jchf.2019.05.016 31521679 PMC7122134

[24] Akhtar KH , Johnston S , Zhao YD , Amil F , Ford L , Lindenfeld J , *et al*. Meta‐analysis analyzing the effect of therapies on 6‐min walk distance in heart failure with reduced ejection fraction. Am J Cardiol 2022;178:72–79. 10.1016/j.amjcard.2022.05.023 35773043

[25] Usman MS , Hamid A , Qazi SU , Kosiborod MN , Bhatt DL , Shahzeb Khan M , *et al*. The effect of SGLT2 inhibitors on health status in patients with heart failure: A systematic review and meta‐analysis. Glob Cardiol 2024;2. 10.4081/cardio.2024.35

[26] Johansson I , Joseph P , Balasubramanian K , McMurray JJV , Lund LH , Ezekowitz JA , *et al*.; G‐CHF Investigators . Health‐related quality of life and mortality in heart failure: The Global Congestive Heart Failure study of 23 000 patients from 40 countries. Circulation 2021;143:2129–2142. 10.1161/CIRCULATIONAHA.120.050850 33906372

[27] Yu C , Hu ZQ , Peng RY . Effects and mechanisms of a microcurrent dressing on skin wound healing: A review. Mil Med Res 2014;1:24. 10.1186/2054-9369-1-24 26000170 PMC4440595

[28] Rame JE , Müller J . Myocardial edema revisited in a new paradigm of cardiac electrical microcurrent application in heart failure. Bioelectricity 2021;3:171–175. 10.1089/bioe.2021.0021 34729463 PMC8558069

[29] Robich MP , Chu LM , Oyamada S , Sodha NR , Sellke FW . Myocardial therapeutic angiogenesis: A review of the state of development and future obstacles. Expert Rev Cardiovasc Ther 2011;9:1469–1479. 10.1586/erc.11.148 22059795 PMC4839193

[30] Haight PJ , Stewart RE , Saarel EV , Pettersson GB , Najm HK , Aziz PF . Lateral thoracotomy for epicardial pacemaker placement in patients with congenital heart disease. Interact Cardiovasc Thorac Surg 2018;26:845–851. 10.1093/icvts/ivx379 29300890

[31] Kalogeropoulos AP , Fonarow GC , Georgiopoulou V , Burkman G , Siwamogsatham S , Patel A , *et al*. Characteristics and outcomes of adult outpatients with heart failure and improved or recovered ejection fraction. JAMA Cardiol 2016;1:510–518. 10.1001/jamacardio.2016.1325 27434402

[32] DeVore AD , Hellkamp AS , Thomas L , Albert NM , Butler J , Patterson JH , *et al*. The association of improvement in left ventricular ejection fraction with outcomes in patients with heart failure with reduced ejection fraction: Data from CHAMP‐HF. Eur J Heart Fail 2022;24:762–770. 10.1002/ejhf.2486 35293088

[33] Savarese G , Vedin O , D'Amario D , Uijl A , Dahlström U , Rosano G , *et al*. Prevalence and prognostic implications of longitudinal ejection fraction change in heart failure. JACC Heart Fail 2019;7:306–317. 10.1016/j.jchf.2018.11.019 30852236

[34] Breathett K , Allen LA , Udelson J , Davis G , Bristow M . Changes in left ventricular ejection fraction predict survival and hospitalization in heart failure with reduced ejection fraction. Circ Heart Fail 2016;9:e002962. 10.1161/CIRCHEARTFAILURE.115.002962 27656000 PMC5082710

