# Supplementary material for: Cardio‐microcurrent device treatment for heart failure with reduced ejection fraction: Results from the C‐MIC II open‐label randomized controlled trial
Source: Eur J Heart Fail. 2025 Jul 15;27(10):1837–49. doi: 10.1002/ejhf.3763 (PMC12575409; doi:10.1002/ejhf.3763)
Supplement: Supplementary file 1 — Appendix S1. Supporting Information. [file EJHF-27-1837-s001.docx]

**Supplementary Appendix**

Table of Contents

[PARTICIPATING SITES AND INVESTIGATORS 2](#_Toc200014821)

[SECTION A: STUDY ADMINISTRATION 4](#_Toc200014822)

[SECTION B: INCLUSION AND EXCLUSION CRITERIA 5](#_Toc200014823)

[Inclusion Criteria 5](#_Toc200014824)

[Exclusion Criteria 5](#_Toc200014825)

[SECTION C: INVESTIGATIONAL DEVICE AND PROCEDURE 8](#_Toc200014826)

[LV Patch Lead 8](#_Toc200014827)

[RV Coil Lead 9](#_Toc200014828)

[Implantable Microcurrent Device 9](#_Toc200014829)

[Portable User Terminal / Mics Adapter 9](#_Toc200014830)

[Procedures involved in the use of the investigational device 10](#_Toc200014831)

[SECTION D: SUPPLEMENTAL TABLES 11](#_Toc200014832)

[Table S1: Visit Schedule 11](#_Toc200014833)

[Table S2: Adverse events Based on the ITT Population 12](#_Toc200014834)

[Table S3: List of Patients with Adverse Events and Corresponding Event Types 13](#_Toc200014835)

[Table S4: Device Deficiencies 14](#_Toc200014836)

[SECTION E: SUPPLEMENTAL FIGURES 15](#_Toc200014837)

[Figure S1: Consort Diagram 15](#_Toc200014838)

[Figure S2: Changes in Peak VO_2_ from baseline to 6 Months 17](#_Toc200014839)

[Figure S3: Changes in Blood Pressure from Baseline to 6 Months 17](#_Toc200014840)

# **PARTICIPATING SITES AND INVESTIGATORS**

| Investigator | Role | Site | Location | Enrollment |
| --- | --- | --- | --- | --- |
| Prof. Dr. Tamara Kovačević Preradović  Dr. Kos Ljiljana  Dr. Unčanin Dragan  Dr. Šobot Nikola  Dr. Živanović Željko  Dr. Dujaković Boris  Dr. Radovanović Zoran | Site PI  Sub-PI  Sub-PI  Sub-PI  Sub-PI  Sub-PI  Sub-PI | University Clinical Centre of Republic of Srpska Banja Luka | Banja Luka, Bosnia & Herzegowina | 22 |
| Prof. Dr. med. Nermir Granov  Dr. Elnur Tahirović | Site PI  Sub-PI | University  Clinical Center of  Sarajevo | Sarajevo, Bosnia & Herzegowina | 5 |
| Prof. MUDr. Petr Neuzil, Ph.D  Milena Prokopova | Site PI  Sub-PI | Department of Cardiology, HNA Homolce Hospital | Prague, Czech Republik | 1 |
| Prof. Dr. Igor Rudez  Prof. Dr. Šime Manola  Dr. Mario Udovičić  Dr. Nikola Pavlović  Dr. Nikola Slišković  Dr. Dubravka Šušnjar  Dr. Marko Kušurin | Site PI  Sub-PI  Sub-PI  Sub-PI  Sub-PI  Sub-PI  Sub-PI | Clinical Hospital Dubrava  Department of Cardiac and Transplant Surgery | Zagreb, Croatia | 2 |
| Prof. Dr. Sasko Jovev  Dr. Vasil Papestiev Dr. Vangiel Zdraveski Dr. Vesna Mitashova Dr. Lazar Kostovski Dr. Marija Gierakaroska Radovikj | Site PI  Sub-PI  Sub-PI  Sub-PI  Sub-PI  Sub-PI | Public Health Institution, University Clinic for State Cardiac Surgery Skopje | Skopje, Republic of North Macedonia | 11 |
| Prof. Dr. Miodrag Peric  Assoc. Prof. Dr. Petar Vuković  Dr. Velibor Ristić  Dr. Ljubomir Đoković  Dr. Nikola Joksić  Dr. Una Radak  Dr. Dragana Košević | Site PI  Sub-PI  Sub-PI  Sub-PI  Sub-PI  Sub-PI  Sub-PI | Institute for Cardiovascular Diseases Dedinje | Belgrade, Serbia | 39 |
| Prof. Dr. Svetozar Putnik  Dr. Ilija Bilbija  Dr. Vojislav Sajić  Assiss. Prof. Dr. Miloš Matkovic  Prof. Dr. Goran Milašinović  Dr. Emilija Nestorović  Prof. Dr. Arsen Ristić | Site PI  Sub-PI  Sub-PI  Sub-PI  Sub-PI  Sub-PI  Sub-PI | Clinical Center of Serbia | Belgrade, Serbia | 2 |
| Prof. Dr. Marija Zdravković  Dr. Predrag Đuran  Dr. Branislava Todić  Dr. Višeslav Popadić  Dr. Slobodan Klašnja  Dr. Jasmina Korica-Trešnjak | Site PI  Sub-PI  Sub-PI  Sub-PI  Sub-PI  Sub-PI | Clinical Hospital Center Bezanijska Kosa | Belgrade, Serbia | 9 |
| Asist. Dr Sci. med. Tanja Popov  Prof. Dr. Aleksandar Redžek  Prof. Dr. Dragan Kovačević  Prof. Dr. Snežana Tadić  Assoc. Prof. Dr. Lazar Velicki  Assist. Prof. Dr. Snežana Bjelić  Dr. Jelena Vučković  Dr. Dragica Andrić  Dr. Andrej Preveden  Dr. Mirko Todić  Mpharm Jasna Radišić-Bosić | Site PI  Sub-PI  Sub-PI  Sub-PI  Sub-PI  Sub-PI  Sub-PI  Sub-PI  Sub-PI  Sub-PI  Sub-PI | Institute of Cardiovascular Diseases, Vojvodina  Clinic for Cardiology | Scremska Kamenica, Serbia | 3 |

# **SECTION A: STUDY ADMINISTRATION**

| Principal Investigator | Jan D. Schmitto, MD  University of Hanover  Hanover, Germany |
| --- | --- |
| Steering Committee | Jesus Eduardo Rame, MD  Thomas Jefferson University  Philadelphia, United Sates  Miodrag Peric, MD  Cardiovascular Institute Dedinje  Belgrade, Serbia  Stefan D. Anker, MD  Charité University  Berlin, Germany  Marat Fudim  Duke University  Durham, United States |
| Data Safety committee | Holger Hotz, MD  Charité University  Berlin, Germany  Michael Laule, MD  Charité University  Berlin, Germany  Johannes Gladitz, PhD  Statistik-Service Dr. Gladitz  Berlin, Germany |
| Sponsor | Berlin Heals, GmbH |
| Echocardiographic Core Laboratory | Martin Kropf, MSc  Imaging Clinical Trial Services  Berlin, Germany  Elisabeth Pieske-Kraigher, MD  Imaging Clinical Trial Services  Berlin, Germany |

# **SECTION B: INCLUSION AND EXCLUSION CRITERIA**

## **Inclusion Criteria**

1. Patients with idiopathic dilative cardiomyopathy who have systolic left ventricular

dysfunction despite of adequate therapy of heart failure (NYHA III – IV

(ambulatory)).

1. Patients with symptomatic chronic heart failure for more than 1 year and less than

5 years at screening.

1. Patients who have a baseline left ventricular ejection fraction of ≥25% and ≤35%

assessed by echocardiography within 30 days prior to study inclusion.

1. Female and male patients aged ≥18 years – 75 years.
2. Patient who understands the nature of the procedure and on-going device therapy.
3. Patient is informed about their participation in a chronic human study and about

the intended treatment period of 6 months which is derived by the fact that according to current knowledge microcurrent treatment exceeding 6 months will

not have additional favorable effects which means will not further improve cardiac

function. Accordingly, battery life is limited. Furthermore, the patient is informed

about the possibility for device explantation, informed regarding possible risks and

is able to give written informed consent prior to any procedures and is considered

willing and able to adhere to study regimen and to return for all follow-up visits.

1. Patients are receiving guideline conform heart failure therapy
2. Patients receiving appropriate, stable guideline conform anti-heart failure therapy

during the 3 months prior study inclusion (OMM). Stable is defined as no more

than a 50% increase or 50% decrease in dose. If the patient is intolerant to full

anti-heart failure medication, documented evidence must be available.

1. Patients who are able to perform a 6-minute walk test.
2. Patients must have a body mass index within the range of 20 - 36 kg/m².
3. Informed consent in writing from patient.
4. Patients with an ICD systems can be included providing:

- Patients are not pacemaker dependent,
- the ICD system uses a single coil electrode,
- the leads can be implanted in such a way that it is ensured, that the metal

parts of the coil electrodes do not touch each other.

## **Exclusion Criteria**

Patients who are not likely to experience improvement of their chronic heart failure by the

micro-current therapy, because the causes of the disease cannot be influenced even if the

patients fulfill the indication for use of the device or if the therapy with the C-MIC System

is not possible or might be associated with unknown risks:

1. Patients who have a potentially correctible cause of heart failure, such as valvular

heart disease or congenital heart disease.

1. Patients with an indication for a CRT system according to current guidelines.
2. Patients who have been hospitalized for heart failure which required the use of

inotropic support within 30 days before enrollment.

1. Patients with systolic blood pressure above 150 mmHg and diastolic blood

pressure above 90 mmHg despite optimal antihypertensive medical treatment.

1. Patients with hemoglobin blood level < 12 g/dl in male and < 10 g/dl in female

patients.

1. Patients with primary pulmonary hypertension
2. Patients who have a genetic connective tissue disease (for example Marfan

syndrome).

1. Patients with constrictive pericarditis.
2. Patients with a prosthetic tricuspid valve.
3. Patients in whom access for implantation of the leads cannot be obtained (i.e.

known venous occlusion, post radiation therapy).

1. Patients who have other preexisting epicardial leads.
2. Patients who have undergone prior heart surgery.
3. Patient with other features (i.e. thorax deformity) that in the eyes of the

investigator make the straightforward placement of the device seem

unlikely.

1. Patients with an ICD system who are pacemaker dependent*
2. Patients with an ICD system with a dual coil electrode.
3. Patients with a CRT system or pacemaker*.
4. Patients with a CCM system*.
5. Current pregnancy or
6. Women of childbearing potential, defined as all women physiologically capable of becoming pregnant, unless they are using highly effective methods of contraception (e.g. intrauterine device, oral contraceptives, barrier methods, or other contraception deemed adequate by the investigator) 2 months before and until 1 month after C-MIC therapy.

Women are considered post-menopausal and not of childbearing potential if they have had 12 months of natural (spontaneous) amenorrhea with an appropriate clinical profile (e.g. age appropriate, history of vasomotor symptoms) or have had surgical bilateral oophorectomy (with or without hysterectomy) or tubal ligation at least 2 months before screening.

1. Breastfeeding/lactating women
2. Patients whose exercise tolerance is limited by a condition other than heart failure

(e.g. chronic obstructive pulmonary disease, peripheral vascular disease,

orthopedic or rheumatologic conditions) or who are unable to participate in a 6-

minute walk test.

1. Patients on immunosuppressive therapy.
2. Patients with present malignancy.
3. Patients with an active infection considered by the investigator to be unsafe for

the patient’s participating in the trial.

1. Patients with renal dysfunction (i.e., estimated glomerular filtration rate

<45 mL/min/ 1,73 m²)

1. Patients with history or presence of relevant liver diseases or hepatic dysfunction

as indicated by abnormal liver function tests at screening and baseline: ALT

(SGPT), AST (SGOT), γ-GT, alkaline, phosphatase and serum bilirubin > 2

× upper limit of normal (ULN). Increase of these liver enzymes caused by

cardiac disorders in the absence of other possible causes of liver damage

are not are not meant by this.

1. Patients with a history of drug or alcohol abuse within the 12 months prior to

screening.

1. Patients who, in the opinion of the Principle Investigator, are unlikely to comply

with the protocol requirements, instructions and trial related restrictions, e.g.,

uncooperative attitude, inability to return for follow-up visits, psychological illness,

and improbability of completing the trial.

1. Participation in any study of an investigational device or drug within 90 days prior

to planned study.

1. Vulnerable Patients (e.g. patients requiring a legal representative, patients kept

in detention, any service within the army, and employees of the sponsor or at an

investigator site).

1. Patients who are not able to avoid the following areas (i.e. due to work):

o Areas with strong magnetic fields

o Areas with strong external electrical influences

o Areas with a warning notice “Access prohibited for pacemaker patients” or

similar.

o Areas with high temperatures

* In case the patient requires a pacemaker or has a CCM device, the impulses given

from the device could change the C-MIC System setting into a safe state and stop

the microcurrent

# **SECTION C: INVESTIGATIONAL DEVICE AND PROCEDURE**

The C-MIC System is a medical device used to treat heart failure per the intended purpose which is to treat heart failure by applying an electrical microcurrent to the heart. The therapy with the C-MIC System is intended to take 6 months. The C-MIC System consists of four parts, of which three are implanted: a transvenous and an epicardial lead and a power source to which the leads are connected. Both leads have the function to transmit microcurrent to the heart. A Portable User Terminal (PUT) is needed for programming and readout of the data recorded by the Implantable Microcurrent Device (IMD). The C-MIC System is *not* a life supporting device, meaning that in case of a “passive” malfunction it imposes no risk to the patient.


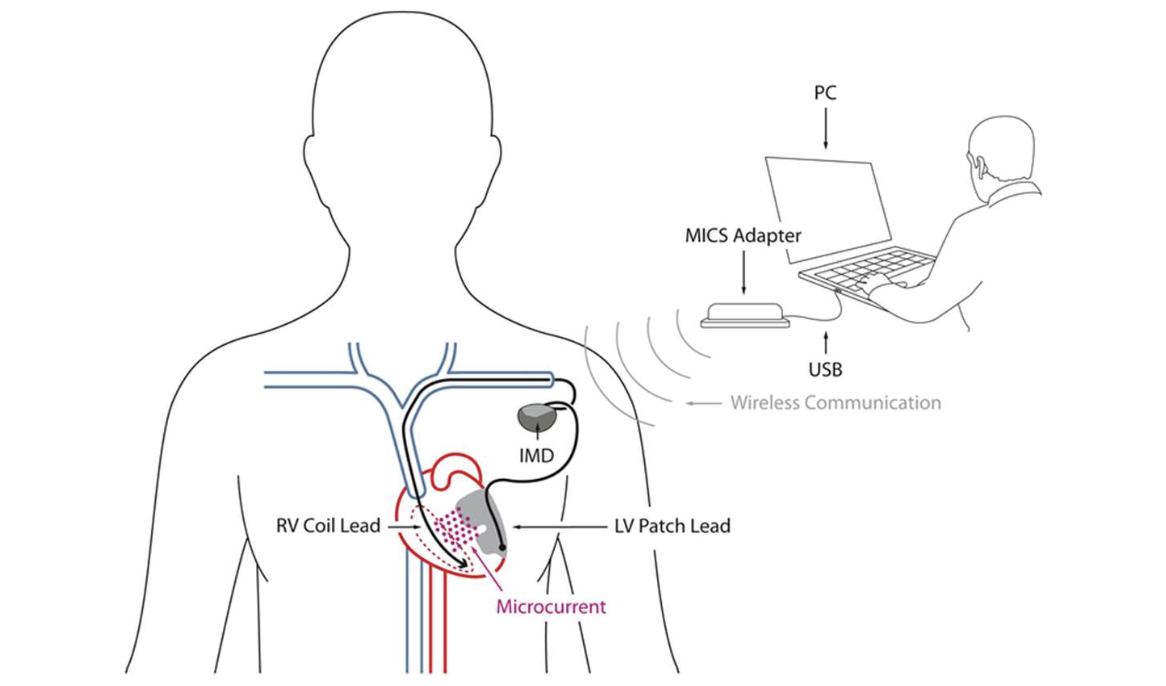

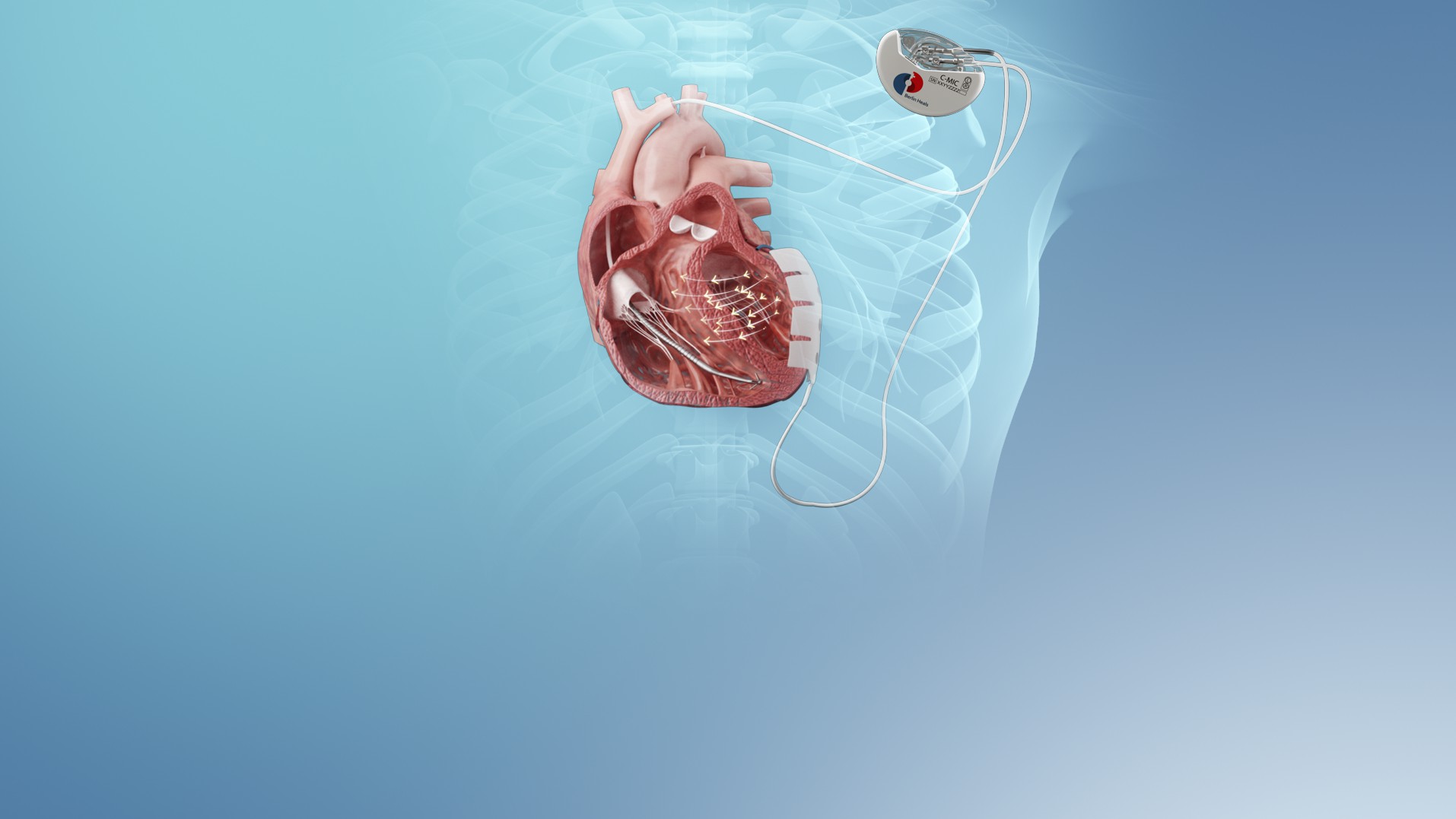


## **LV Patch Lead**

The LV patch lead is secured to the pericardium with its conductive side facing the LV free wall, delivering microcurrent alongside the endocardial electrode. It features a platinum/iridium (Pt 90/Ir 10) mesh and an insulated conductive line with an IS-1 connector, similar to early ICD patch leads (Gollob and Seger, 2001).


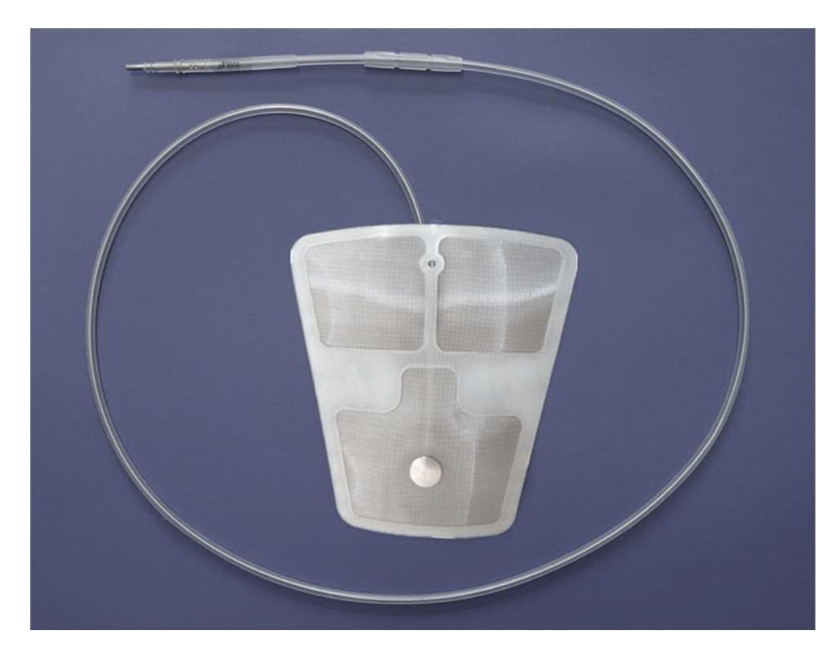


## **RV Coil Lead**

The RV Coil Lead is transvenously placed in the right heart and passively anchored with tines, serving as the counter electrode. Its design resembles single-coil defibrillator leads (Sticherling et al. 2011), featuring an electrically active Pt(90)/Ir(10) surface in the right heart. The conductor line and IMD connection are identical to the LV patch electrode.


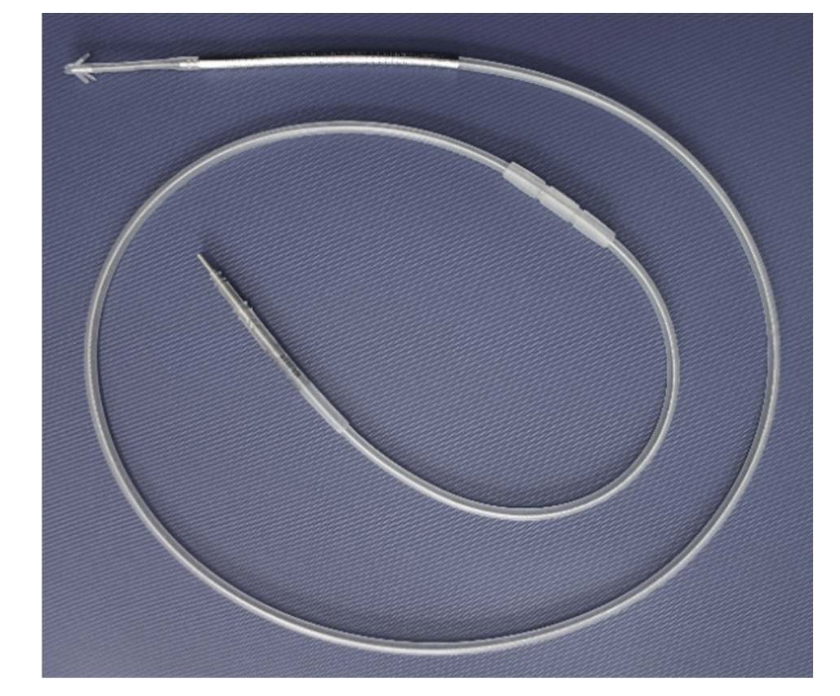


## **Implantable Microcurrent Device**

The Implantable Microcurrent Device (IMD) powers the C-MIC System, resembling a pacemaker but generating continuous direct microcurrent in the μ-ampere range, independent of heart activity. Made of Titanium Grade 2 with an Epoxy Epotek 301 header, it is typically placed subcutaneously in the pectoral region. Both leads connect via an IS-1 plug. Microcurrent parameters have default settings but can be adjusted through the Portable User Terminal (PUT), which also records and reads therapy parameters and error messages.


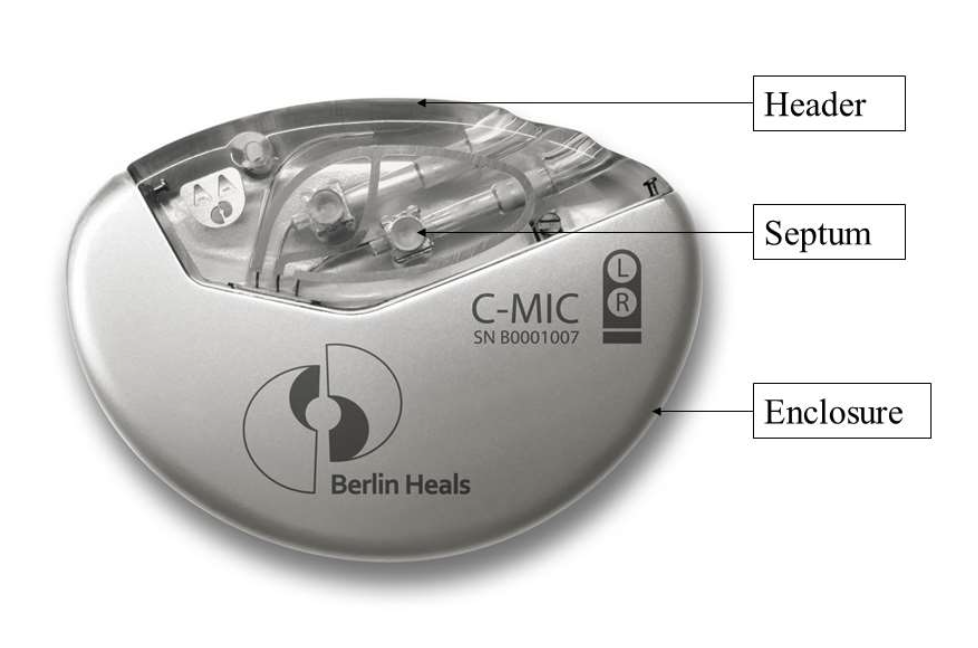


## **Portable User Terminal / Mics Adapter**

The Portable User Terminal (PUT) is a computer-based platform with a MICS adapter for wireless IMD communication. It features a graphical interface (PUT-GUI) for user interaction. Before implantation, a self-test ensures proper IMD function. The PUT starts or pauses microcurrent therapy, reads stored data, and adjusts settings or resets error modes.

## **Procedures involved in the use of the investigational device**

The transvenous lead is implanted via the Seldinger technique, while the epicardial lead is placed through a small thoracic incision and secured to the pericardium. Once positioned, both leads are tunneled subcutaneously to the infraclavicular region and connected to the power source, which is placed in a subcutaneous pocket. The implantation process includes an IMD *Self-Test* before surgery, the procedure itself, and a *Connection Check* before wound closure to ensure proper system function. C-MIC therapy begins one day post-implantation once the patient is stable. Device function is regularly monitored using a no-touch technique, with data logged pseudonymized for study purposes and personalized for hospital records. The system is turned off at the final study visit, and removal is optional if indicated or desired.

# **SECTION D: SUPPLEMENTAL TABLES**

## **Table S1: Visit Schedule**


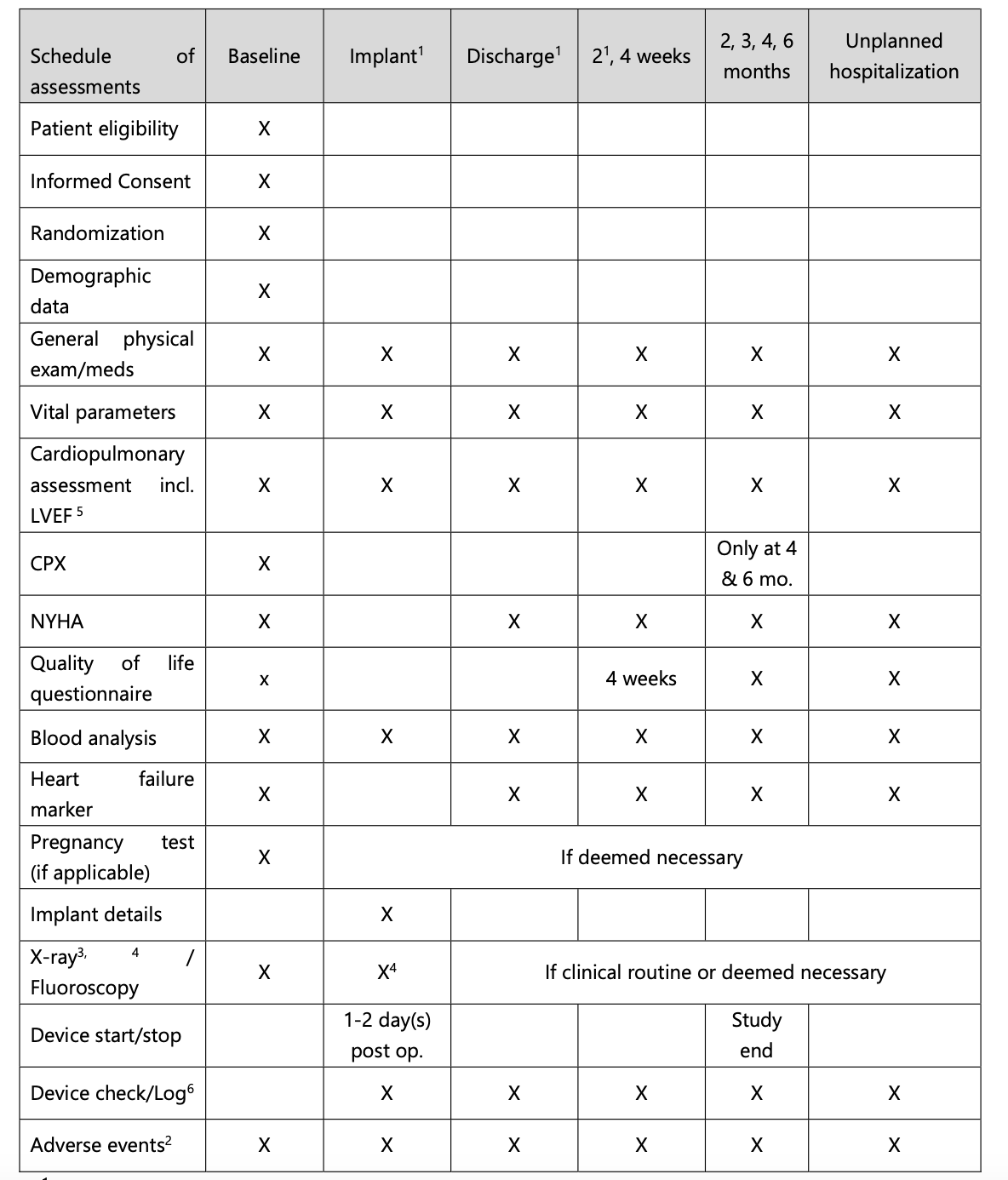


^1^Only in C-MIC Group, no assessment of control group at these timepoints.

^2^ AEs will be followed at all times

^3^ Imaging according to local standard practice, may be included to baseline data

collection if performed within 90 days prior to implant

^4^ Imaging can also be performed with echocardiography

^5^ Additional Core Lab evaluation at baseline, week 4, month 4 and month 6

^6^ Wireless data readout: Check state of device and download pseudonymized IMD

protocol.

## **Table S2: Adverse events Based on the ITT Population**

|  |  | Device Group  (N=35) | | Control Group  (N=35) | | Overall  (N=70) | |
| --- | --- | --- | --- | --- | --- | --- | --- |
| Parameter | Characteristic | n (pat) | n (event) | n (pat) | n (event) | n (pat) | n (event) |
| Adverse events | N (%) | 15 (42.9) | 22 | 6 (17.1) | 6 | 21 (30.0) | 28 |
| Relationship to surgical procedure | causal | 2 (5.7) | 2 | - | - | 2 (2.9) | 2 |
|  | not related | 6 (17.1) | 13 | N/A | N/A | 12 (17.1) | 19 |
|  | Possible | 5 (14.3) | 5 | - | - | 5 (7.1) | 5 |
|  | probable | 2 (5.7) | 2 | - | - | 2 (2.9) | 2 |
| Relationship to the C-MIC system | Not related | 12 (34.3) | 19 | N/A | N/A | 18 (25.7) | 25 |
|  | possible | 2 (5.7) | 2 | - | - | 2 (2.9) | 2 |
|  | probable | 1 (2.9) | 1 | - | - | 1 (1.4) | 1 |
| Expectedness | AE expected | 6 (17.1) | 6 | 1 (2.9) | 1 | 7 (10.0) | 7 |
|  | AE unexpected | 2 (5.7) | 2 | - | - | 2 (2.9) | 2 |
|  | Not applicable | 10 (28.6) | 14 | 5 (14.3) | 5 | 15 (21.4) | 19 |
| Severity | Mild | 10(28.6) | 16 | 5 (14.3) | 5 | 15 (21.4) | 21 |
|  | Moderate | 1 (2.9) | 1 | 1 (2.9) | 1 | 2 (2.9) | 2 |
|  | Severe | 4 (11.4) | 5 | - | - | 4 (5.7) | 5 |
| Device deficiency |  |  | - | - | - |  | - |
| SAE |  | 5 (14.3) | 6 | 1 (2.9) | 1 | 6 (8.6) | 7 |

## **Table S3: List of Patients with Adverse Events and Corresponding Event Types**

| Group^*^ | Type | SAE | Procedure Related | Device Related |
| --- | --- | --- | --- | --- |
| Device – Patient 1 | Hypotension | Yes | No | No |
| Device – Patient 2 | inflammation of the wound | No | Yes | No |
| Device – Patient 3 | haemothorax | Yes | Yes | No |
| Device – Patient 3 | worsening of renal function | Yes | No | No |
| Device – Patient 4 | haemothorax | Yes | Yes | No |
| Device – Patient 5 | wound infection 4 months after c-  mic implantation | No | Yes | Yes |
| Device – Patient 6 | Covid 19 | No | No | No |
| Device – Patient 6 | Burning and tingling sensation in left  hemithorax (near to left sternal side) | No | Yes | Yes |
| Device – Patient 7 | Pericardial effusion | No | Yes | Yes |
| Device – Patient 8 | Pericardial effusion, Tamponade Pleural  effusion in left hemithorax | Yes | Yes | No |
| Device – Patient 9 | Ishiadical pain. | No | No | No |
| Device – Patient 10 | Covid 19 | No | No | No |
| Device – Patient 11 | Covid 19 | No | No | No |
| Device– Patient 11 | Ventricular arrhythmia | Yes | No | No |
| Device – Patient 11 | Bronchopneumonia | No | No | No |
| Device – Patient 11 | Loss of appetite and vomiting | No | No | No |
| Device – Patient 12 | Pain in the left knee | No | No | Np |
| Device – Patient 13 | Swelling and tenderness. | No | Yes | No |
| Device – Patient 14 | Non-sustained ventricular tachycardia | No | No | No |
| Device – Patient 15 | Dizziness | No | No | No |
| Device – Patient 15 | Dry cough | No | No | No |
| Device – Patient 15 | Non-sustained ventricular tachycardia | No | No | No |
| Control – Patient 1 | Fatigue and shortness of breath | No | N/A | N/A |
| Control – Patient 2 | Elevated body temperature | No | N/A | N/A |
| Control – Patient 3 | leg fracture | Yes | N/A | N/A |
| Control – Patient 4 | Twisted ankle | No | N/A | N/A |
| Control – Patient 5 | Ventricular extrasystoles | No | N/A | N/A |
| Control – Patient 6 | Respiratory infection. | No | N/A | N/A |
| ^*^Some patients experienced multiple adverse events | | | | |

## **Table S4: Device Deficiencies**

| Parameter | Characteristic | Device Deficiency (N=19)^*^ |
| --- | --- | --- |
| Nature of the problem | Malfunction | 2 (10.5) |
|  | Other system error | 4 (21.1) |
|  | Missing | 13 (68.4) |
| Were any actions taken to resolve the problem? | No | 1 (5.3) |
|  | Yes | 18 (94.7) |
| Component involved – C-MIC System | Yes | 19 (100) |
| Component involved – IMD | No | 6 (31.6) |
|  | Yes | 13 (68.4) |
| Component involved – LV patch lead | No | 15 (78.9) |
|  | Yes | 4 (21.1) |
| Component involved – RV coil lead | No | 15 (78.9) |
|  | Yes | 4 (21.1) |
| Did this device deficiency involve the patient | No | 17 (89.5) |
|  | Yes | 2 (10.5) |
| Was the patient’s safety or well-being impacted by the device deficiency? | No | 19 (100) |
| Could the device deficiency potentially affect the patient's safety or well-being in the future? | No | 19 (100) |
| All reported deficiencies were software-related and resolved by simply restarting the device. As a built-in safety feature, the device automatically shuts off in response to unusual voltage fluctuations. During follow-up visits, sites routinely verified device functionality and downloaded technical logs. If the device was found in “error mode,” it was restarted. None of these events were associated with clinical symptoms or disruptions in therapy delivery, except in one patient with an ICD, where therapy remained off for an extended period. | | |

# **SECTION E: SUPPLEMENTAL FIGURES**

**Figure S1: Consort Diagram**. Among the control group, exclusions from the Per-Protocol (PP) analysis population were due to: one patient with poor-quality echocardiographic imaging at the 6-month visit, one patient whose 6-month visit occurred outside the allowable window (>244 days), one patient later found to be ineligible due to not meeting the heart failure history criterion (within 1–5 years prior to randomization), and one patient who withdrew from the trial after the 3-month visit. In the device group, two patients were excluded from the PP population because they were retrospectively found to be ineligible, having not met the heart failure history criterion (1–5 years).


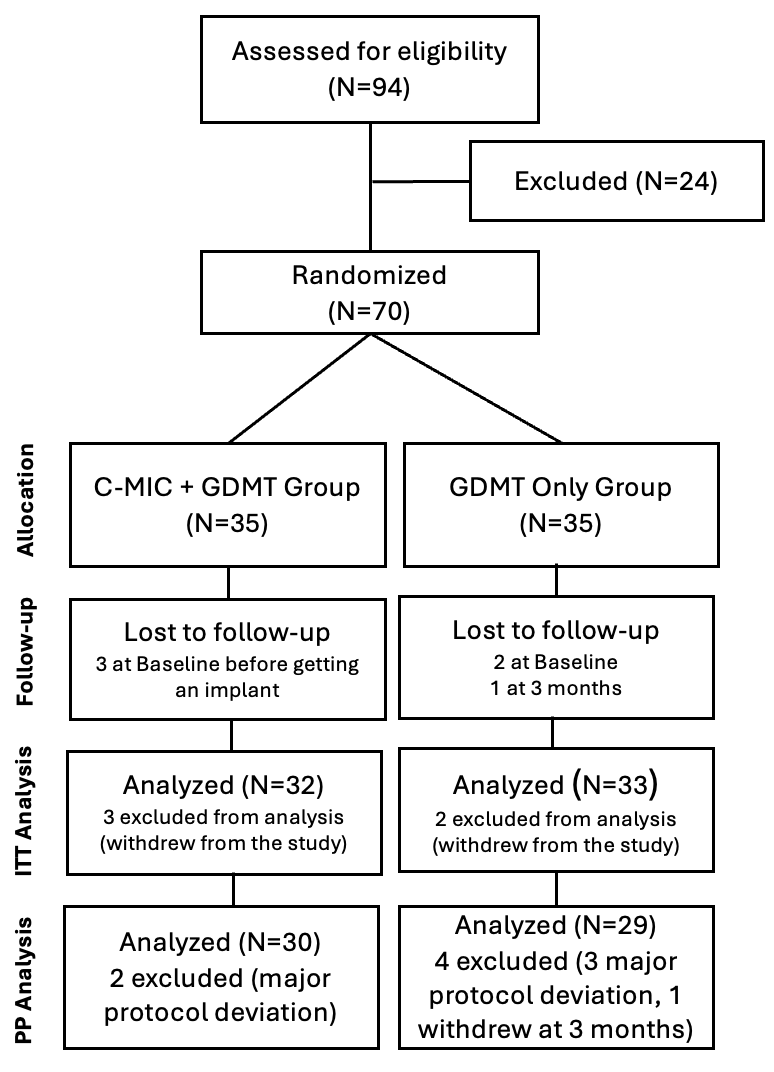


##

## **Figure S2: Changes in Peak VO2 from baseline to 6 Months.**


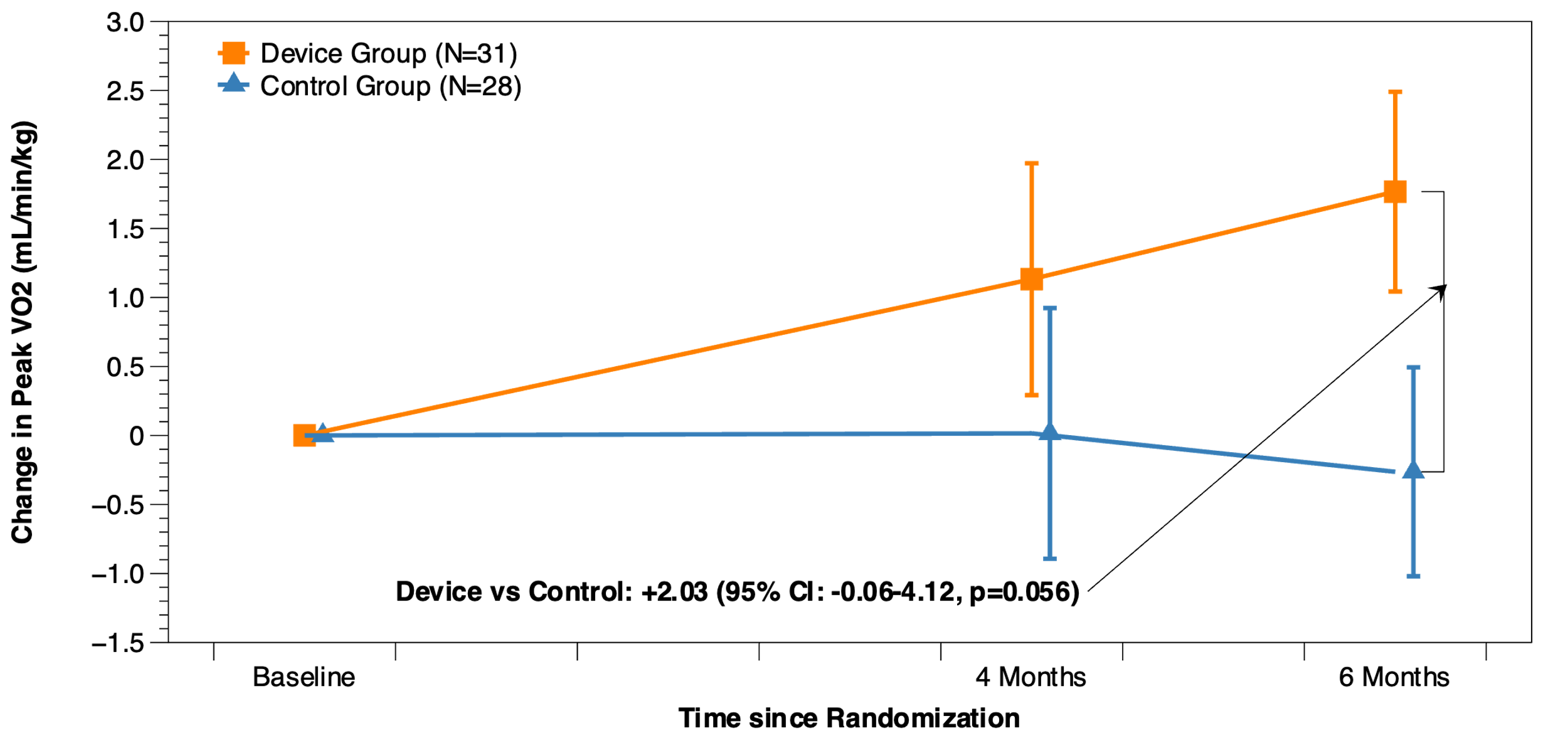


**Figure S3: Blood Pressure at Each Time Point (a) and Change from Baseline to 6 Months (b).** Blood pressure was adequately controlled in both the device and control groups, with no significant difference observed in the change from baseline to 6 months.


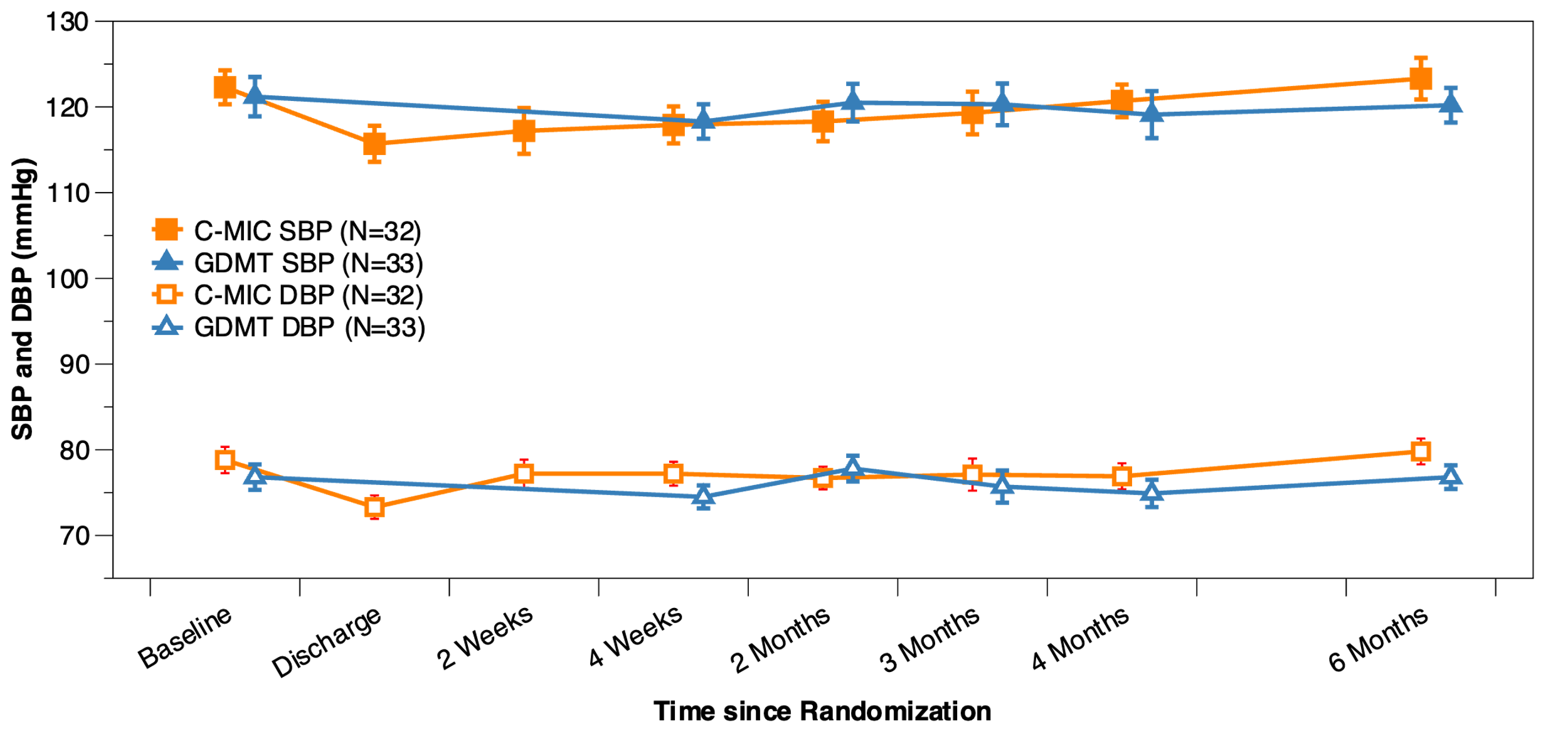


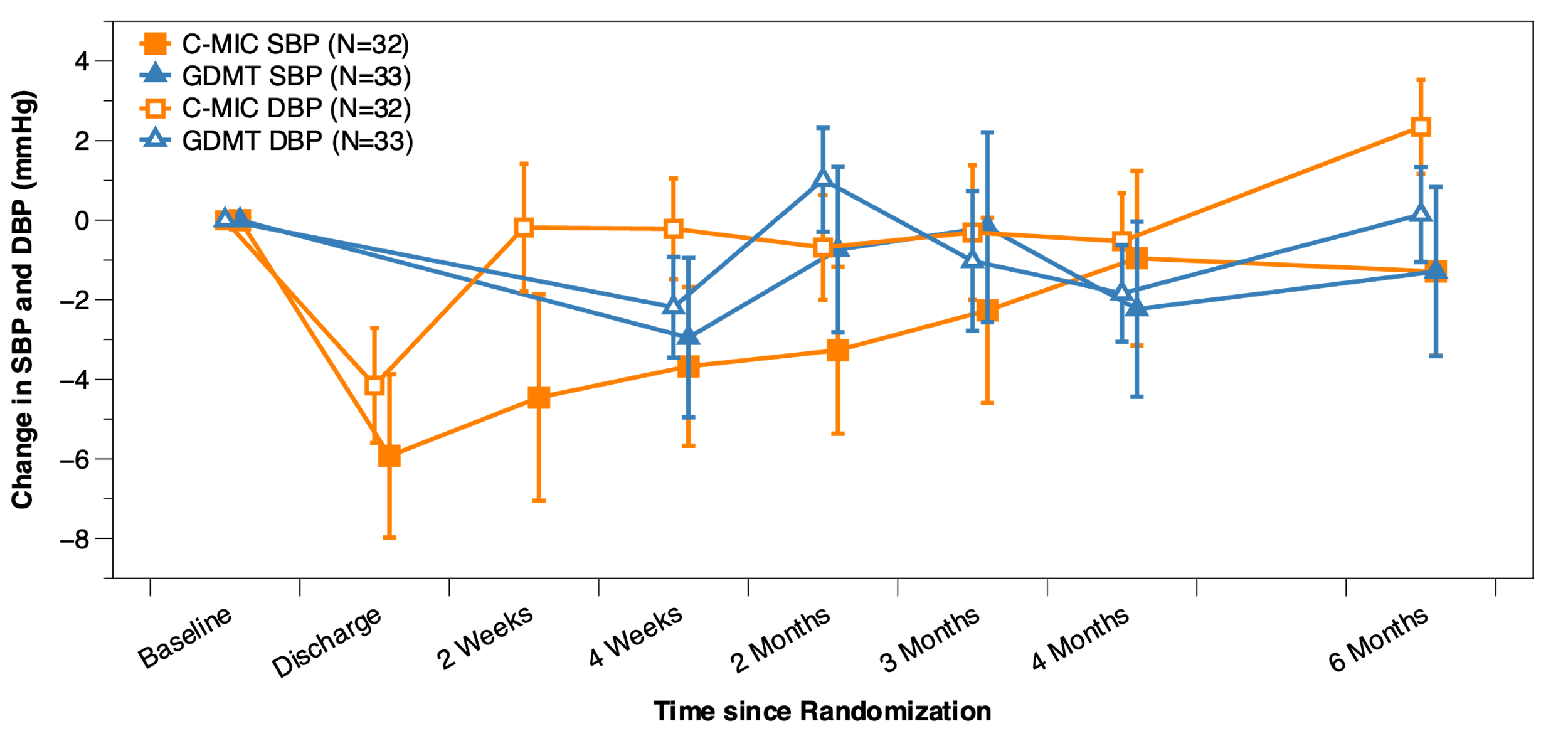


**REFERENCES**

1. Gollob, Michael H., and John J. Seger. 2001. “Current Status of the Implantable Cardioverter- Defibrillator.” *CHEST Journal* 119 (4): 1210–1221.
2. Sticherling, Christian, Markus Zabel, Sebastian Spencker, Udo Meyerfeldt, Lars Eckardt, Steffen Behrens, Michael Niehaus, and for the ADRIA Investigators. 2011. “Comparison of a Novel, Single-Lead Atrial Sensing System With a Dual-Chamber Implantable Cardioverter-Defibrillator System in Patients Without Antibradycardia Pacing Indications Results of a Randomized Study.” *Circulation: Arrhythmia and Electrophysiology* 4 (1): 56–63.
